# Supplementary material for: Conceptualization of Biomimicry in Engineering Context among Undergraduate and High School Students: An International Interdisciplinary Exploration
Source: Biomimetics (Basel). 2023 Mar 17;8(1):125. doi: 10.3390/biomimetics8010125 (PMC10046154; doi:10.3390/biomimetics8010125)
Supplement: Supplementary file 1 [file biomimetics-08-00125-s001.zip › biomimetics-2218765-supplementary.pdf]

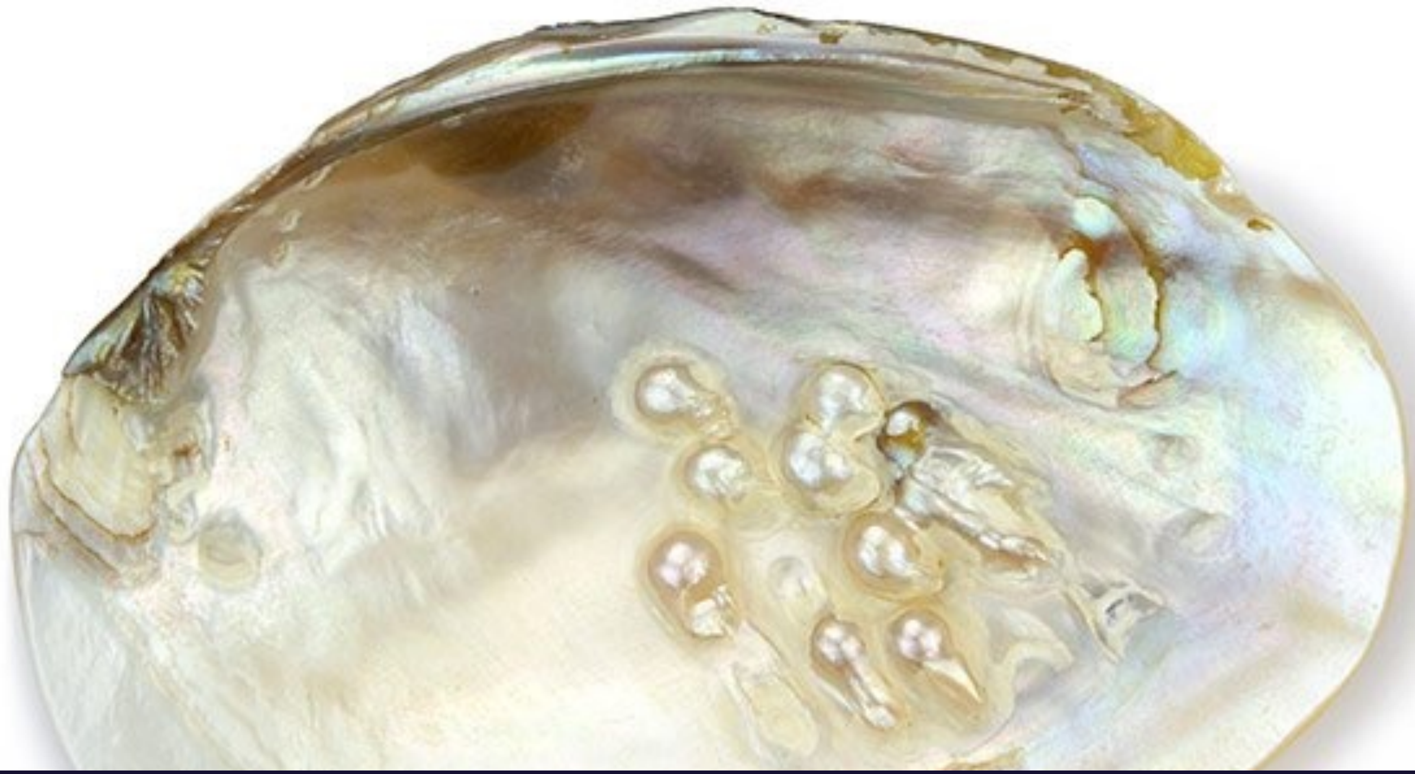

Taking inspiration from Nature to engineer tomorrow's world

May 2022

NANYANG TECHNOLOGICAL UNIVERSITY SINGAPORE

Prof Hortense

Welcome to NTU!

Taking inspiration from Nature to engineer tomorrow's world

Take a seat and write your name.

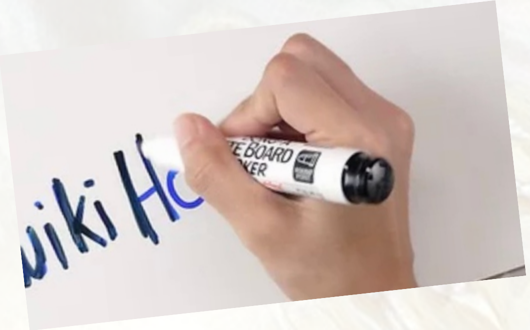

Answer the surveys if not done so already.

Welcome to NTU

School of Mechanical and Aerospace Engineering

Thank you for filling the 2 pre-surveys sent by email earlier.

Nanyang Technological University

NANYANG TECHNOLOGICAL UNIVERSITY SINGAPORE

30

1991-2021

Celebrating its 30 anniversary

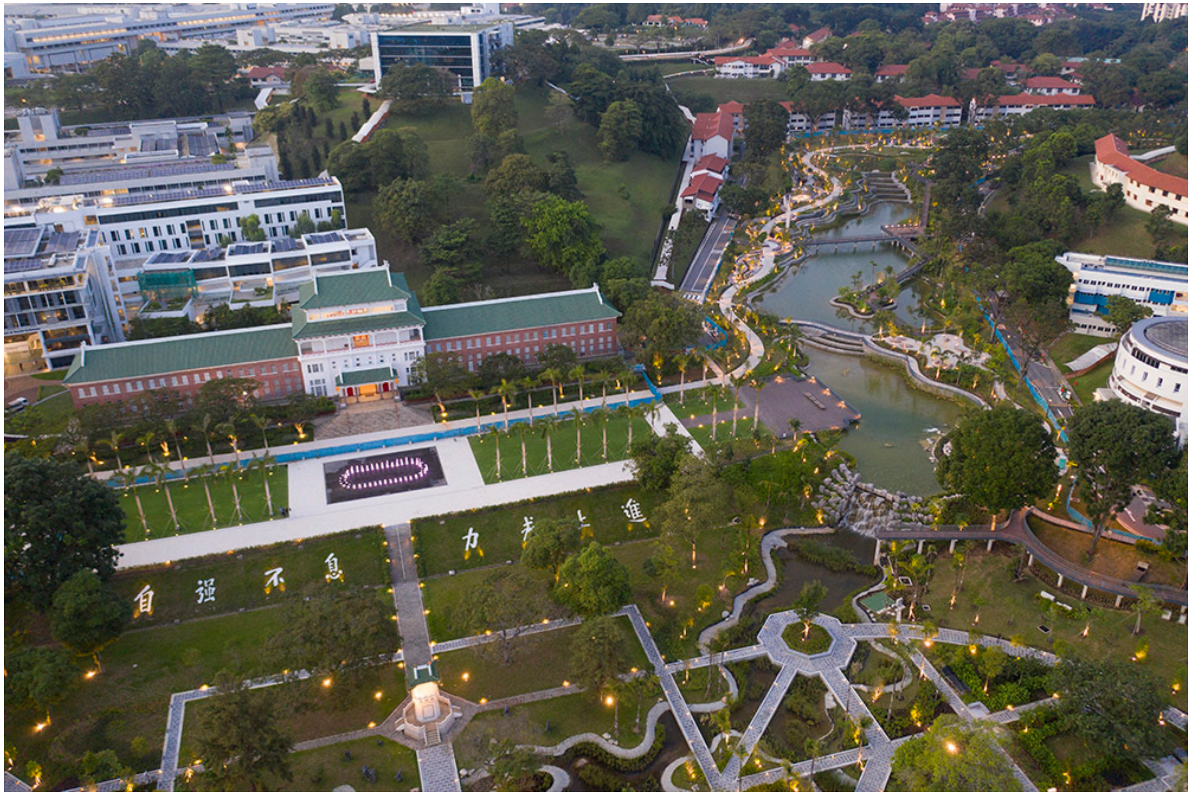

School of MAE
‘Laboratory for dense and multifunctional composites’.

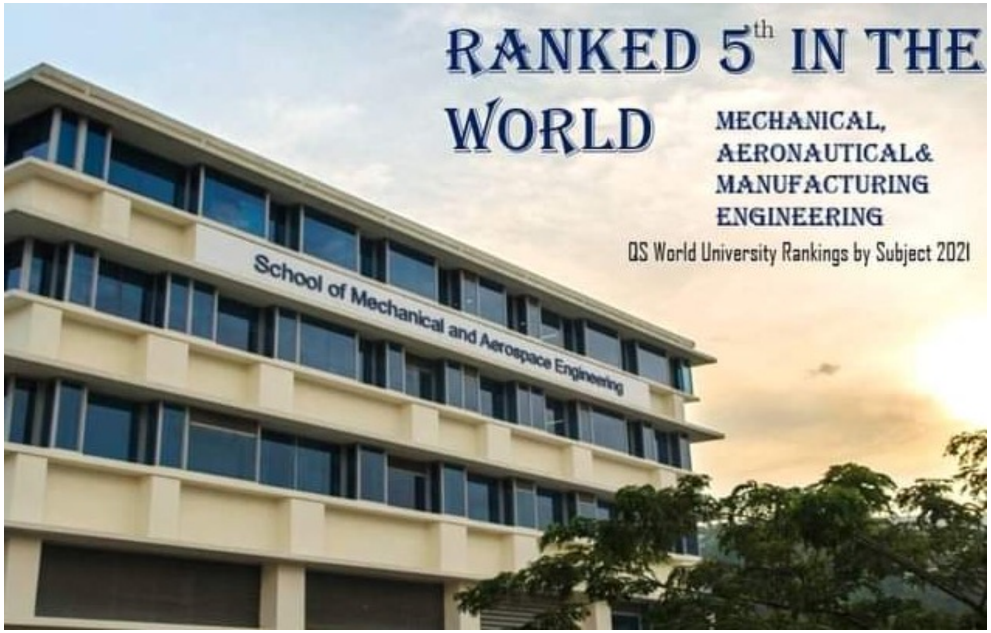

- Additive manufacturing of ceramics and composites
- Bioinspired materials
- Multifunctional and structural (strong/hard) materials

Hortense@ntu.edu.sg
http://www.ntu.edu.sg/home/hortense

NANYANG TECHNOLOGICAL UNIVERSITY SINGAPORE

Taking inspiration from Nature to engineer tomorrow’s world

Objectives

- Learn what is bioinspiration and biomimicry
- How to apply bioinspiration in practice
- Discover examples from the research
- Discuss and share ideas with peers

Taking inspiration from Nature to engineer tomorrow's world

Outline of the module
9:30 – 1 pm

- Introduction on biomimetics and bioinspiration (ca. 1 hr)
- Be a bioinspired engineer: lab activity (ca. 1 hr)
- Share what you learned and imagined: communication activity (ca. 1.15 hr)
- Concluding words

Wooclap

WEB

1 Connect to www.wooclap.com/SEENTU

2 You can participate

Pictures and laptop to prepare 5 slides for sharing to the class.

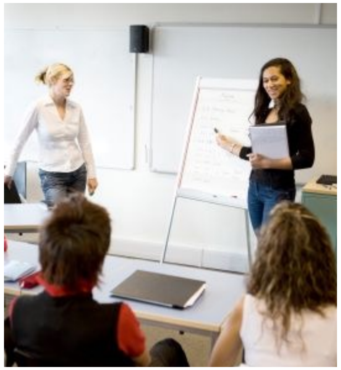

Envelop and biomaterial for lab activity

Facilitators

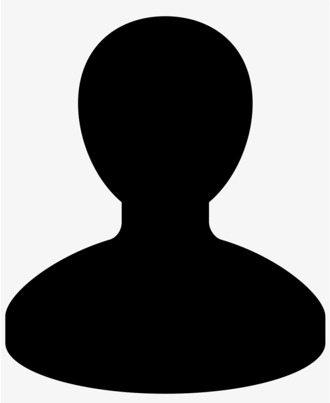

(Edamame)

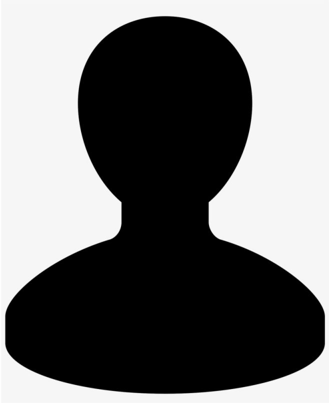

(Lotus)

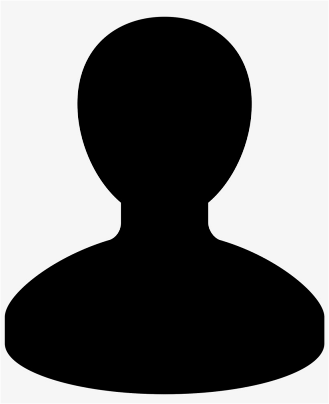

(Seashell)

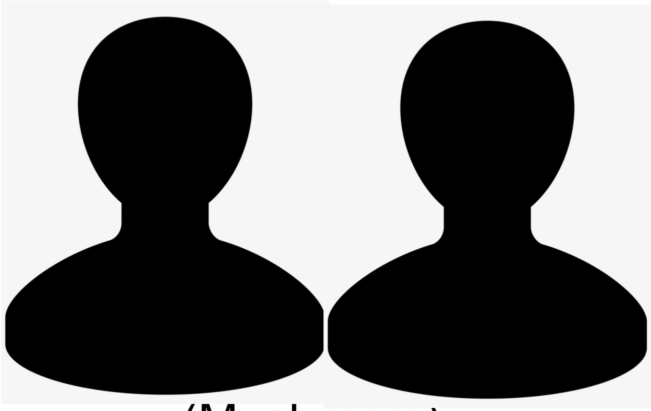

(Mushroom)

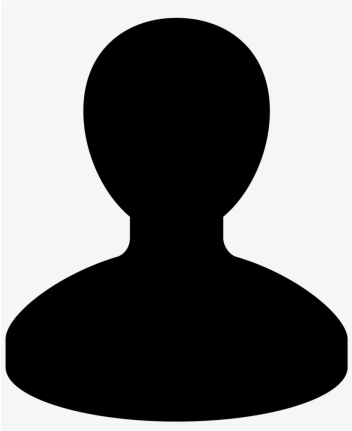

(General facilitator)

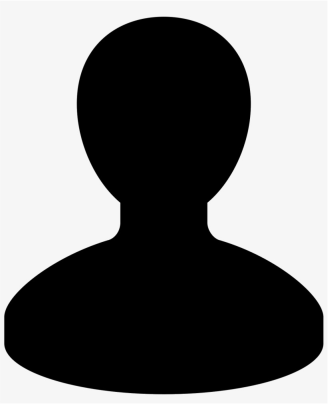

(Peach gum)

NANYANG TECHNOLOGICAL UNIVERSITY SINGAPORE

Biomimicry?

Biomimicry

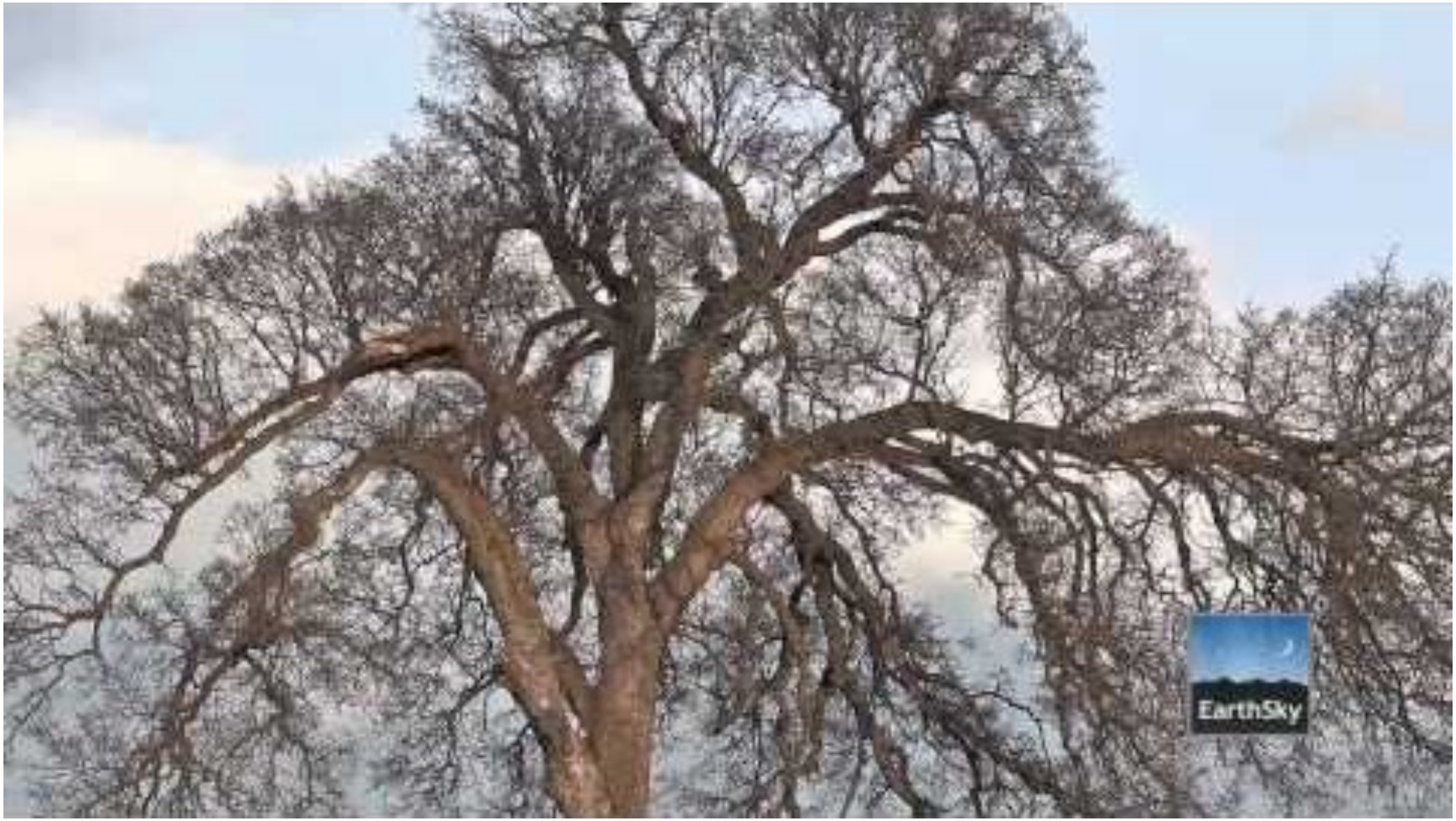

Biomimicry

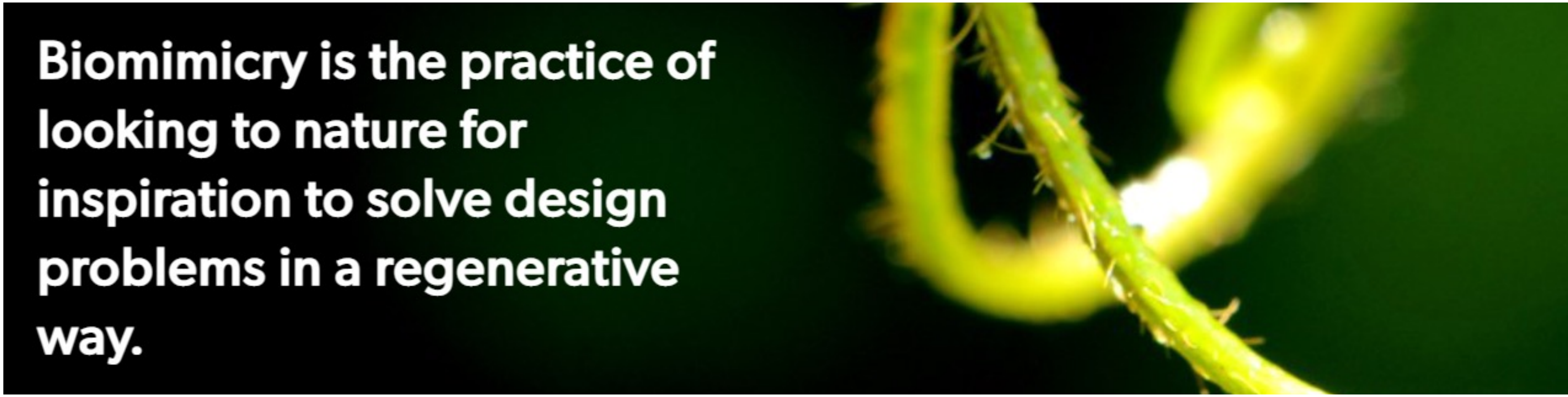

BIOMIMICRY INSTITUTE

2006

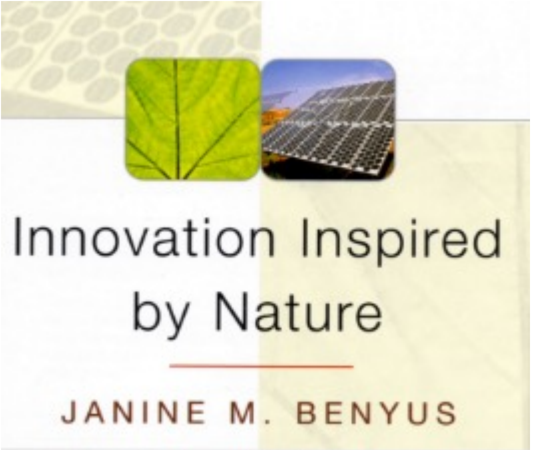

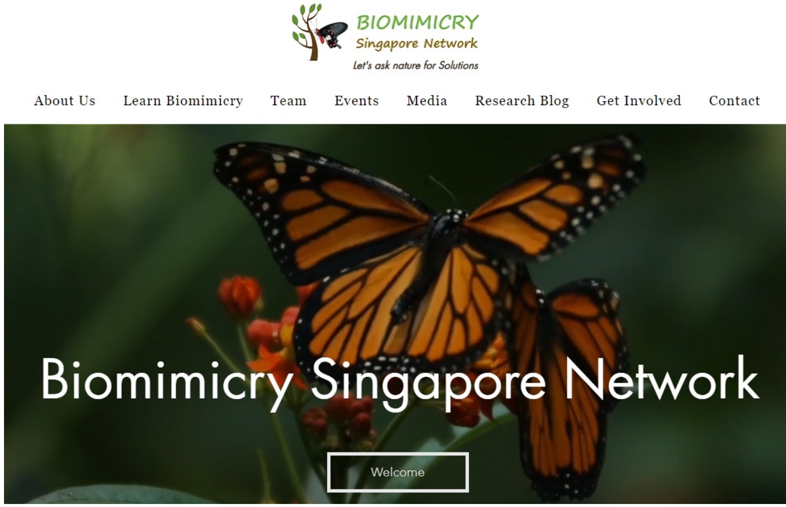

NANYANG TECHNOLOGICAL UNIVERSITY SINGAPORE

Biomimicry

Leonardo Da Vinci 15th century

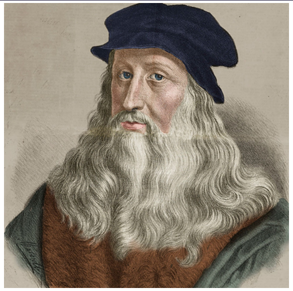

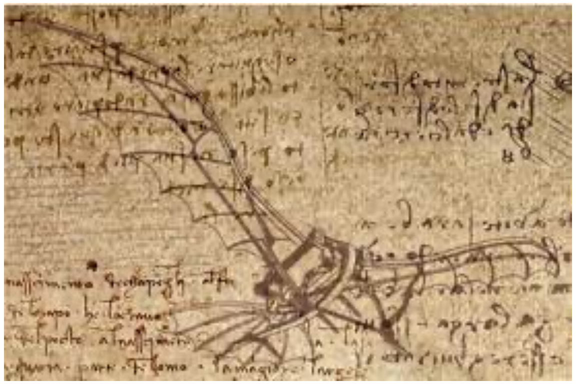

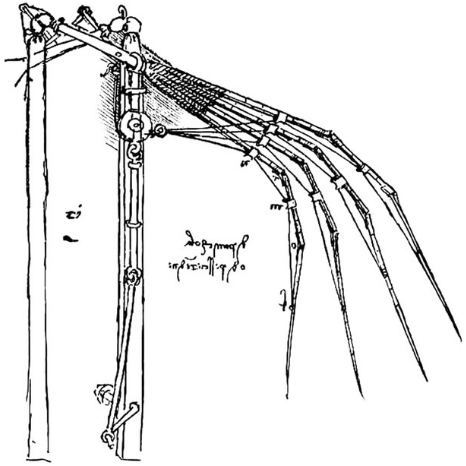

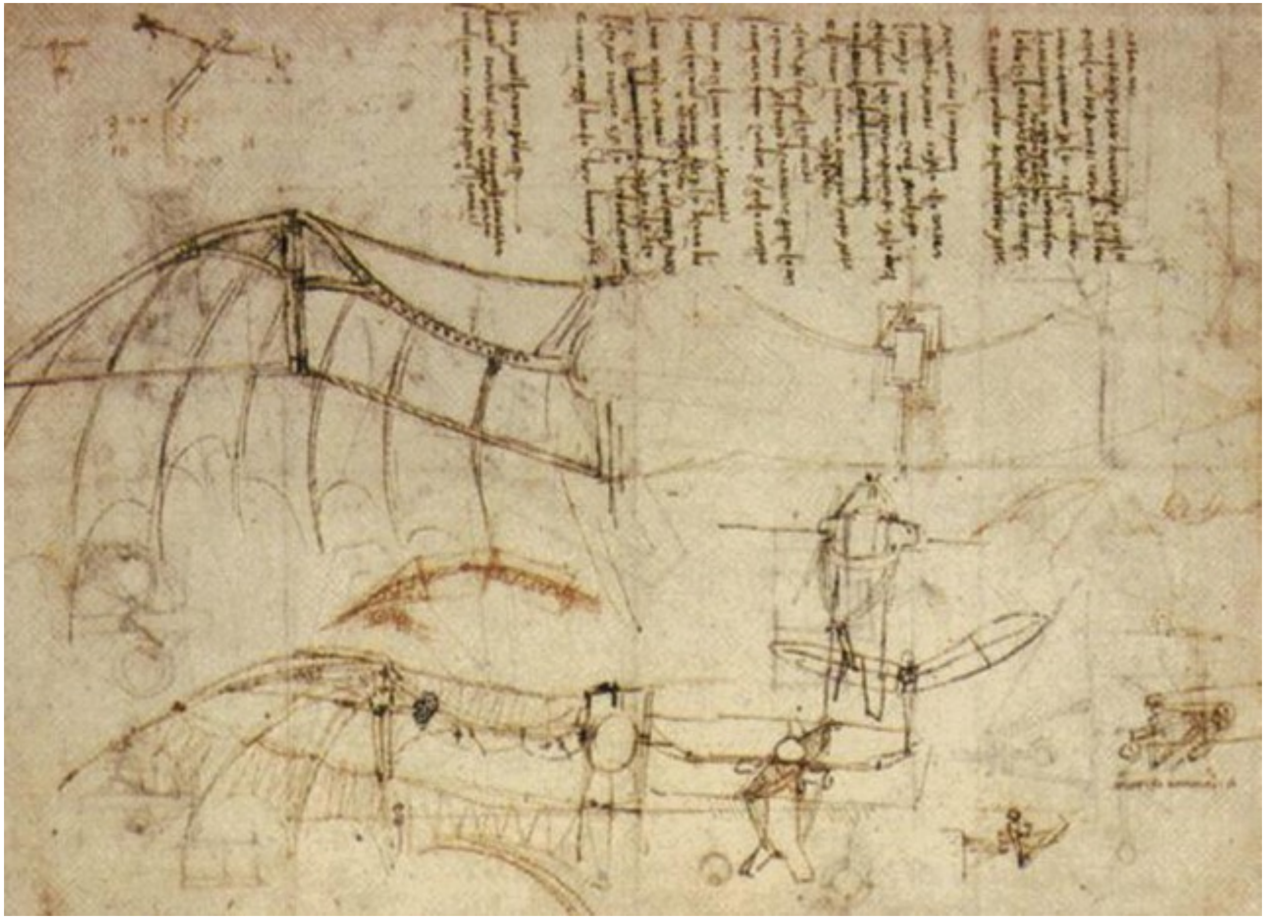

NANYANG TECHNOLOGICAL UNIVERSITY SINGAPORE

Biomimicry – activity 1

Terms, topics associated to biomimicry?
Where can we find biomimicry around us?
Why can it be useful?

www.wooclap.com/SEENTU

Mindmap that is multidisciplinary, from theory to application, in all domains

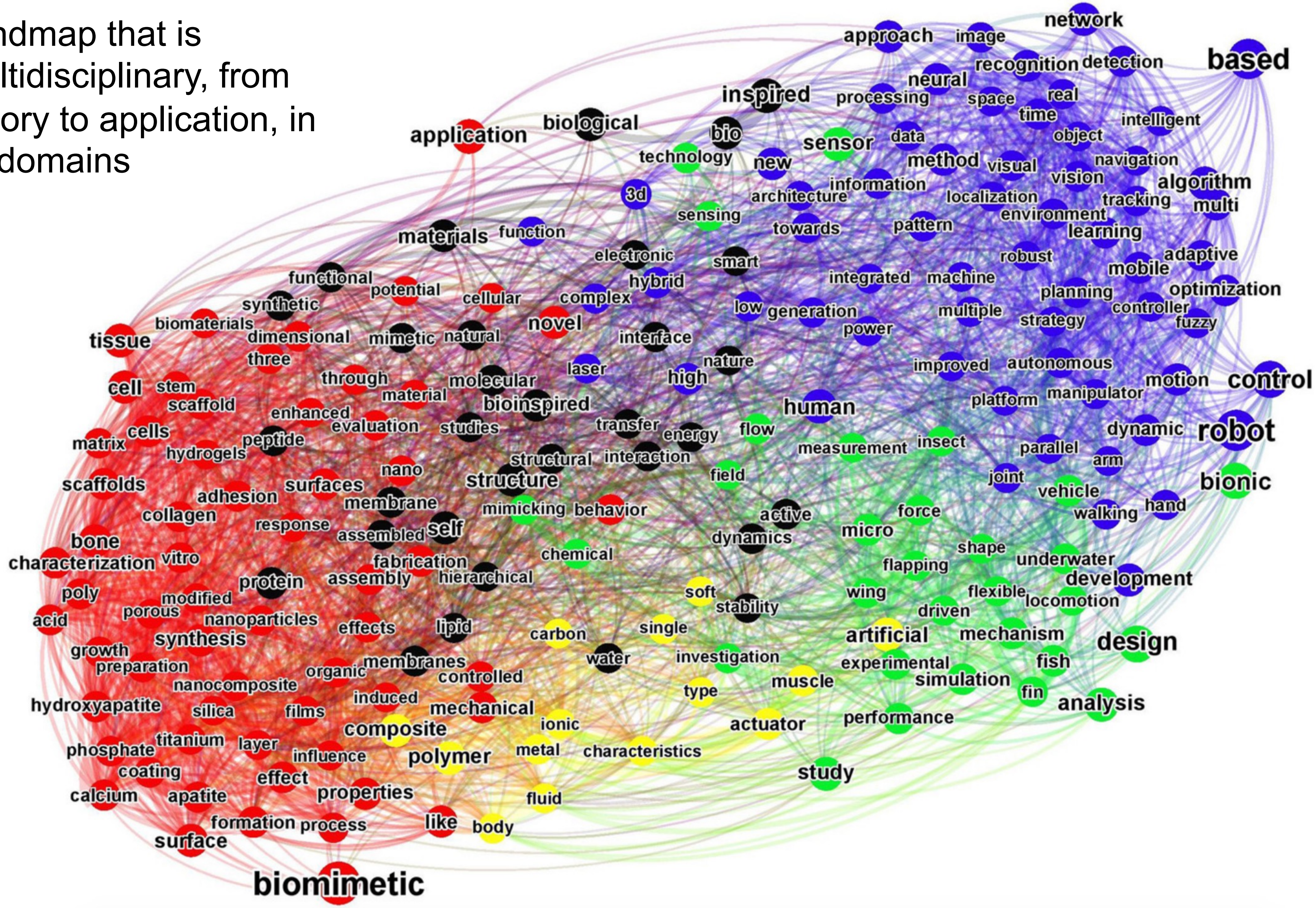

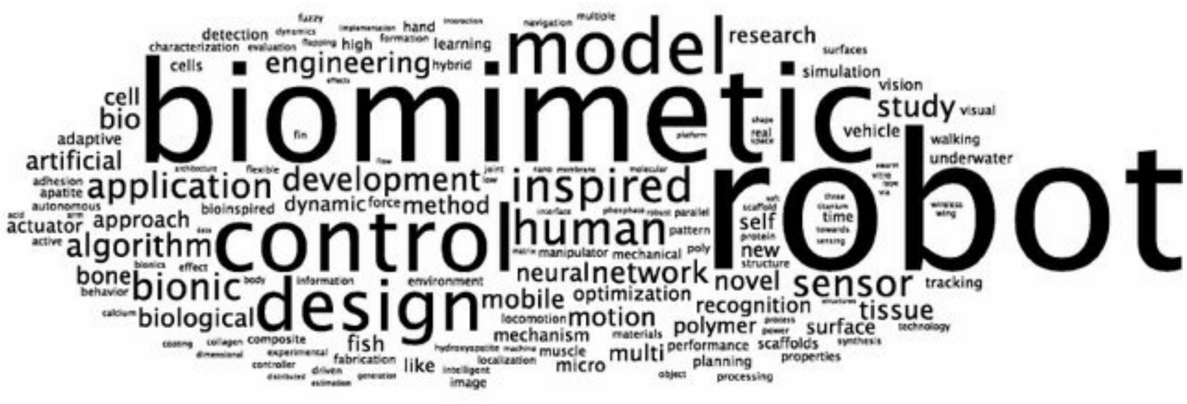

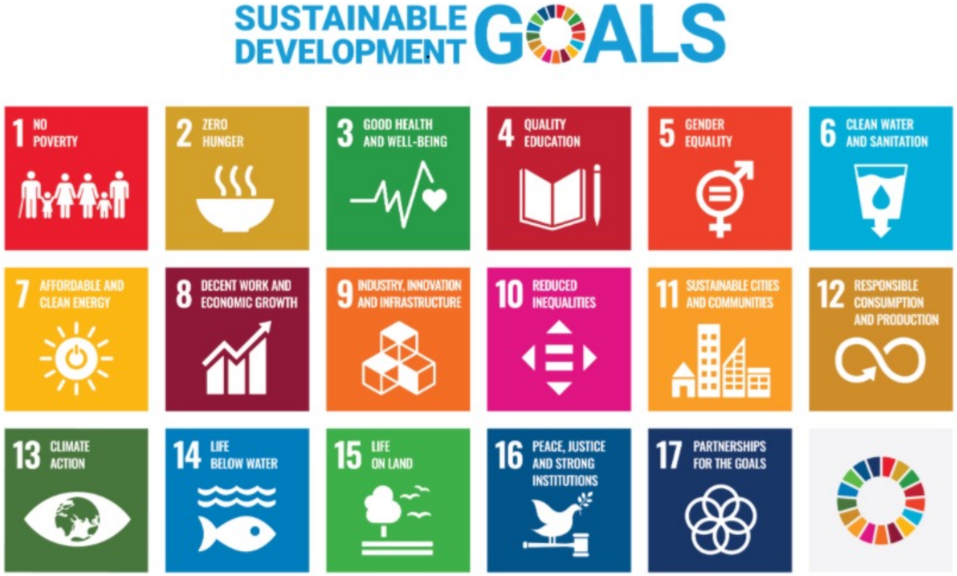

Bioinspiration to be one strategy to solve today’s grand challenges.

Figure 1. Bioinspired Innovation's Forecasted Impact on GDP in 2030

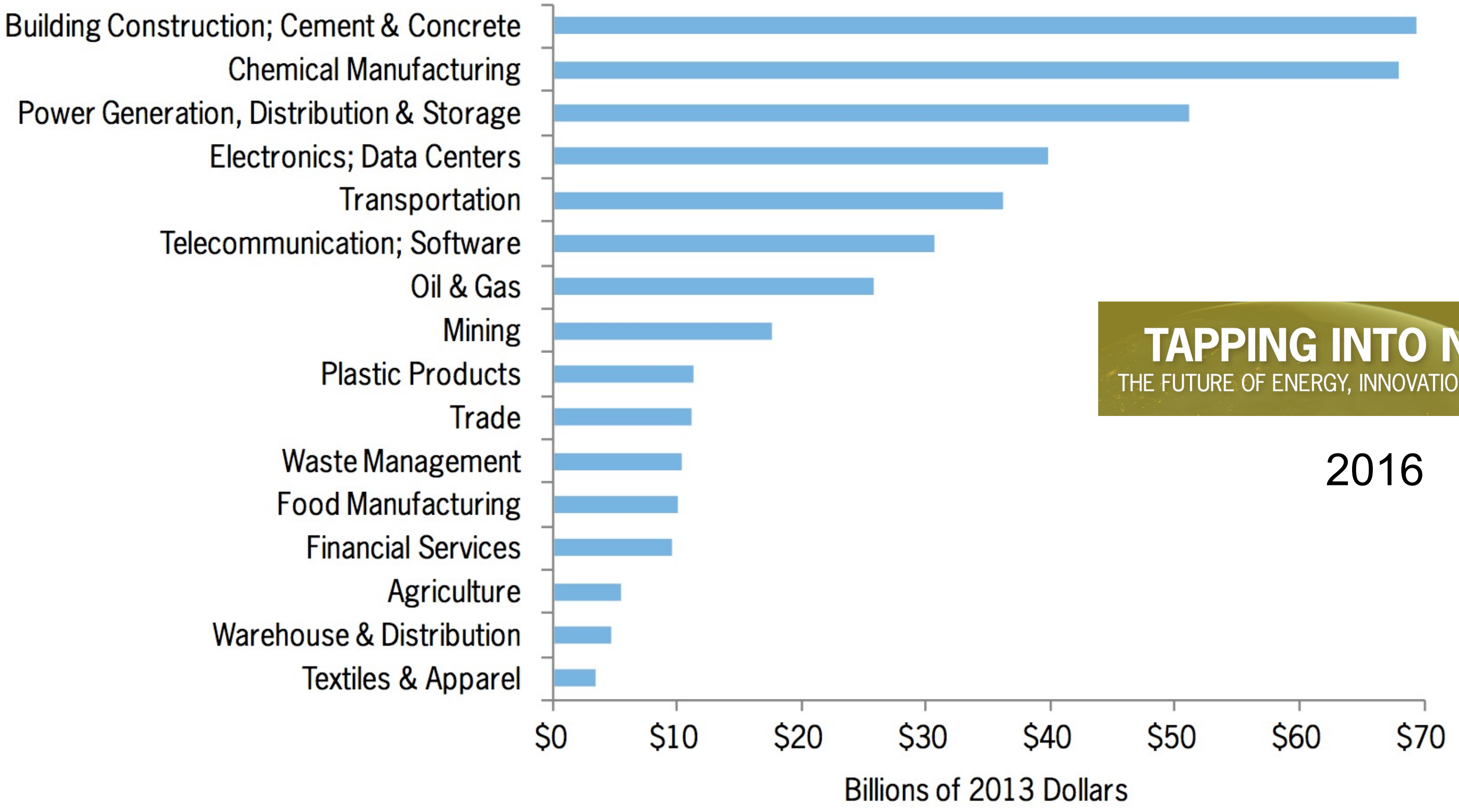

Source: FBEI

TAPPING INTO NATURE
THE FUTURE OF ENERGY, INNOVATION, AND BUSINESS

2016

Increased employment in bioinspired engineering.

NANYANG TECHNOLOGICAL UNIVERSITY SINGAPORE

Bioinspiration?

Bio-inspiration goes beyond biomimicry.

It is not copying Nature, but **understanding** the key mechanisms and **applying** them to solve our engineering problems.

(eg: there is no example of space shuttles in Nature)

The principle of applying bio-inspiration – activity 2

1. Determining the problem to solve/ aims.

E.g. hot Earth to cold space

2. Identifying a natural system where the problem is solved and understanding the concepts

E.g. a species that lives in hot and cold

3. Developing a method to apply this solution to the engineering problem.

E.g. apply to different ranges of temperature

The principle of applying bio-inspiration – activity 2

Let’s look at some examples

Example in architecture

(1) Problem/ aims

-Energy Efficiency
-Insulated greenhouse
-Isolation of indoor and outdoor environment
- Natural lighting

(2) Natural system

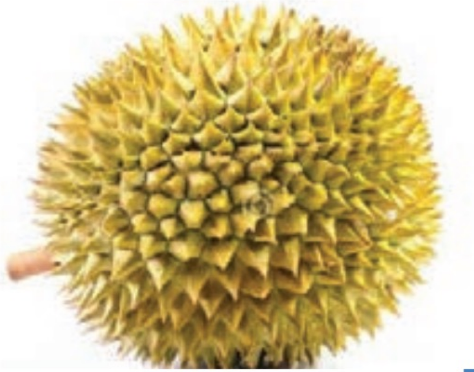

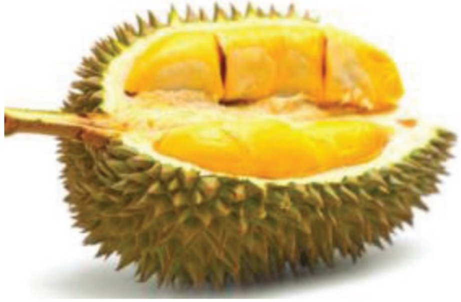

(3) Apply

-Energy reduction by 30%
-Capturing solar energy
-Reduction of artificial lighting by 55%

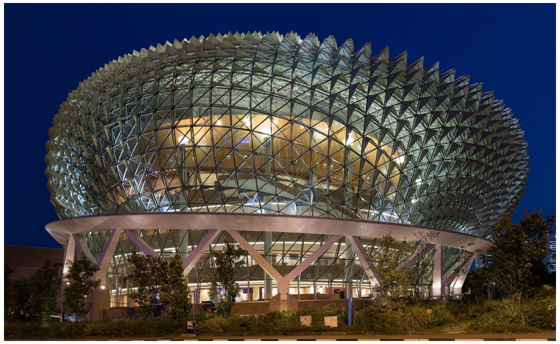

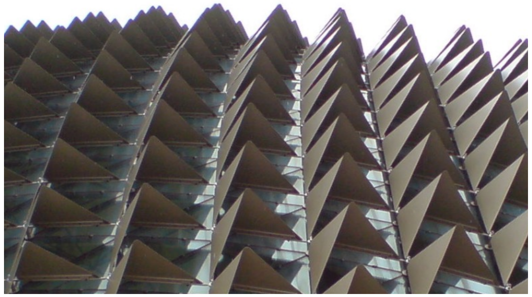

Example in transportation

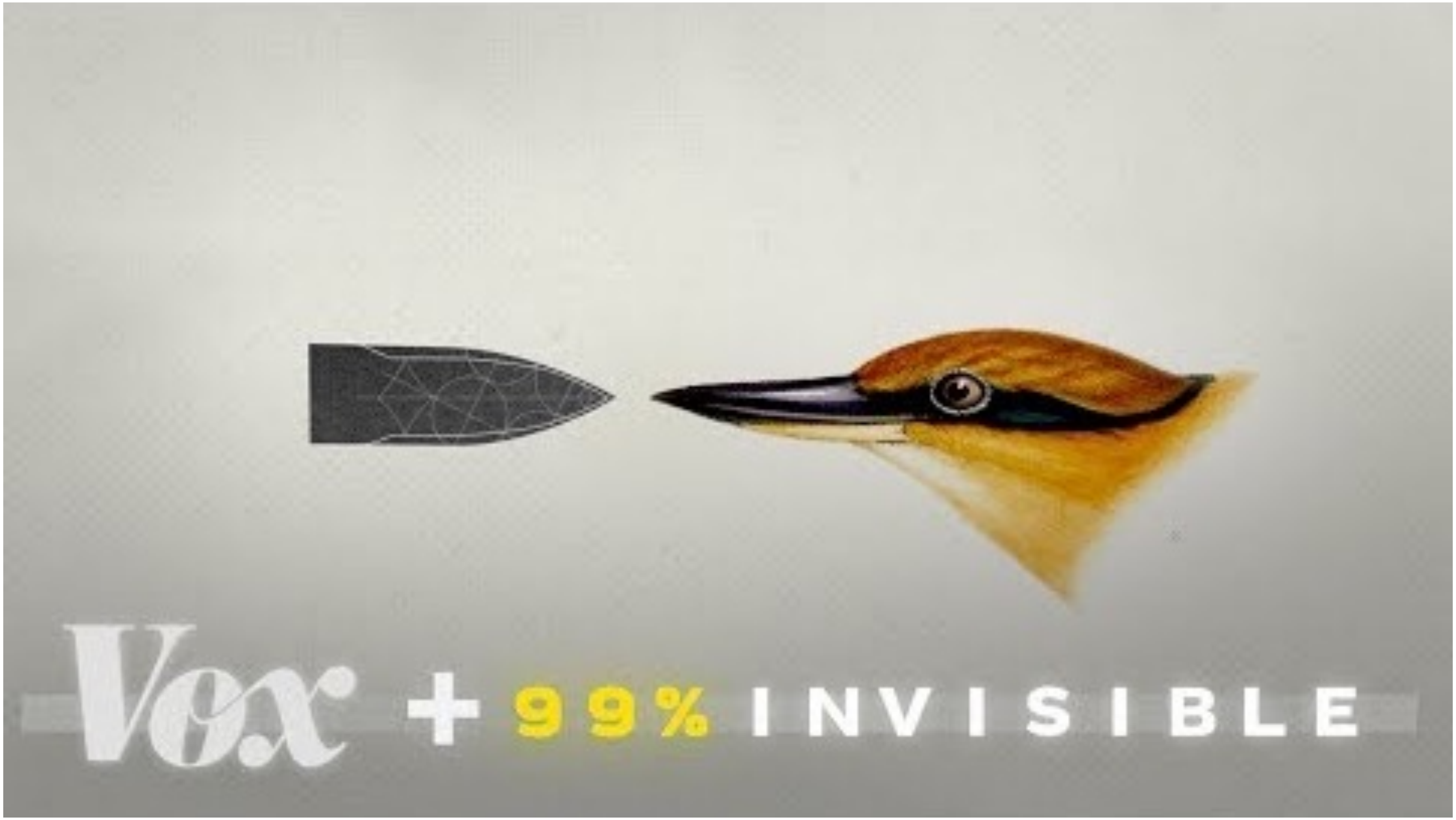

Stop at 2:08

Example in transportation

(1) Problem/ aims

(2) Natural system

(3) Apply

www.wooclap.com/SEENTU

3 - 5 min

Example in transportation

(1) Problem/ aims

(2) Natural system

(3) Apply

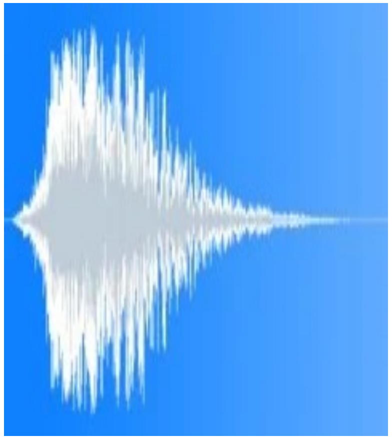

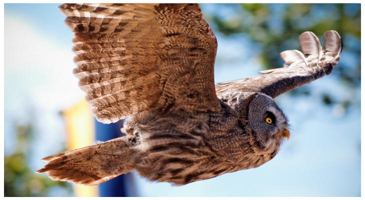

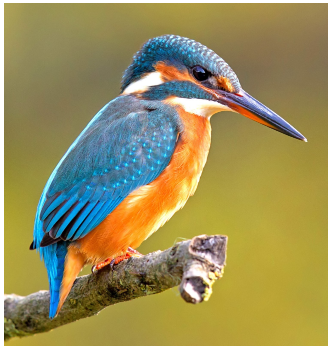

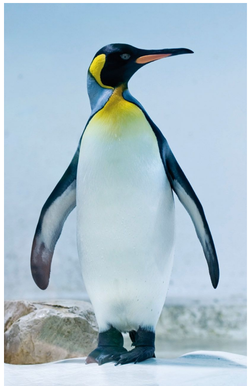

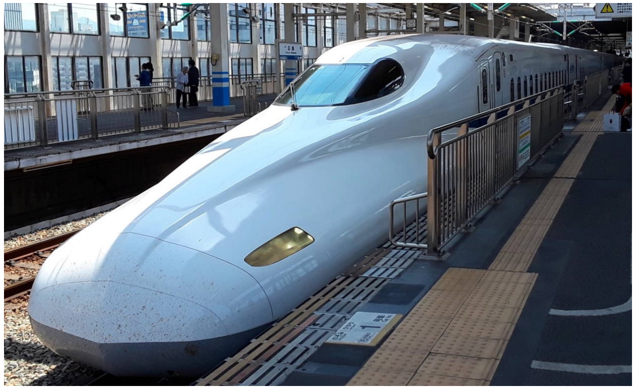

Fast and
low noise

Feathers, belly
shape, and
beak of birds.

New design shapes

Achieve: 10% faster,
15% less electricity, and
below the sound limit

Example in water treatment (desalination)

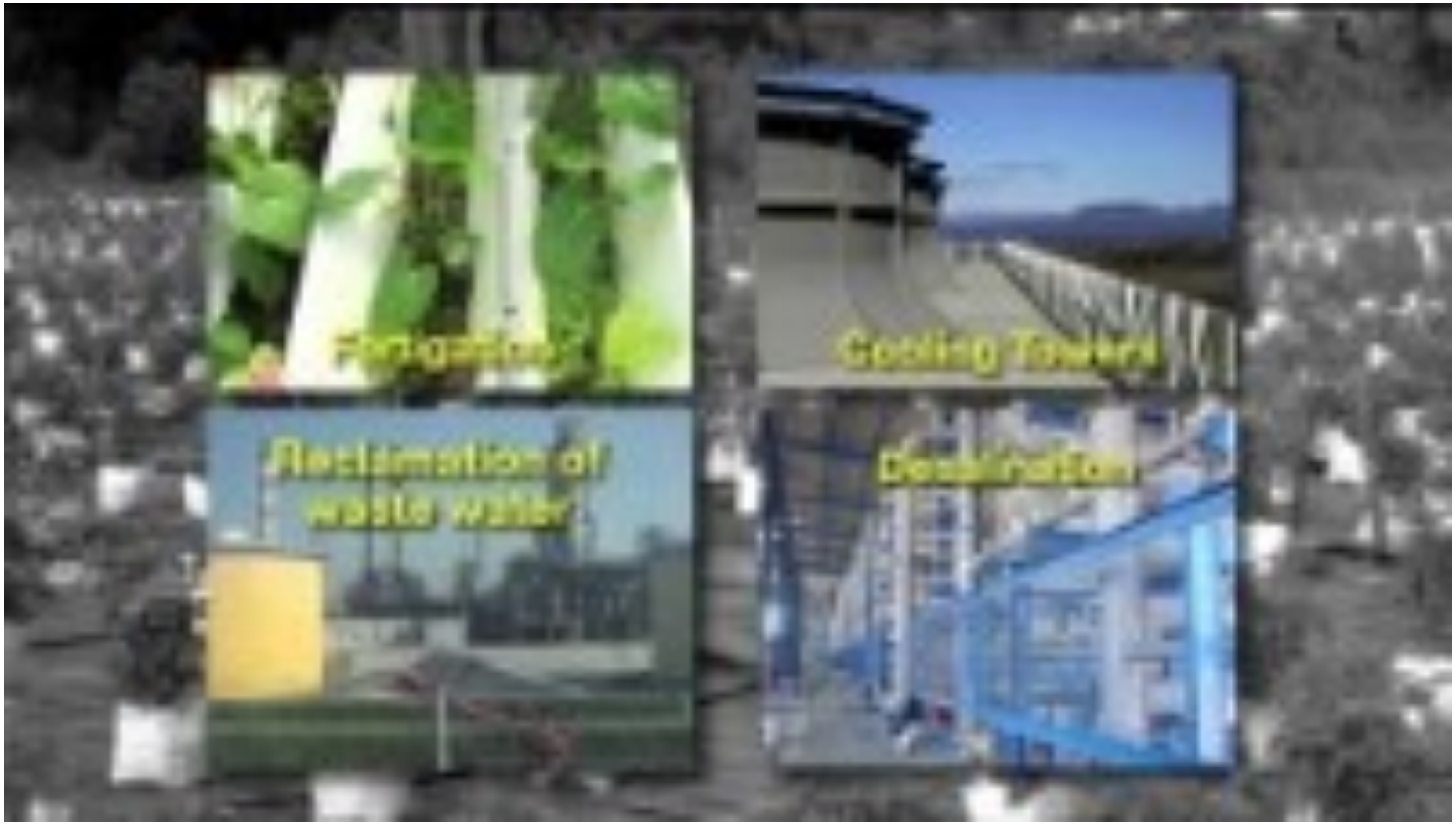

Stop at 1:23

AQUAPORIN

NANYANG TECHNOLOGICAL UNIVERSITY SINGAPORE

Example in water treatment (desalination)

(1) Problem/ aims

(2) Natural system

(3) Apply

www.wooclap.com/SEENTU

NANYANG TECHNOLOGICAL UNIVERSITY SINGAPORE

3-5 min

Example in water treatment (desalination)

(1) Problem/ aims

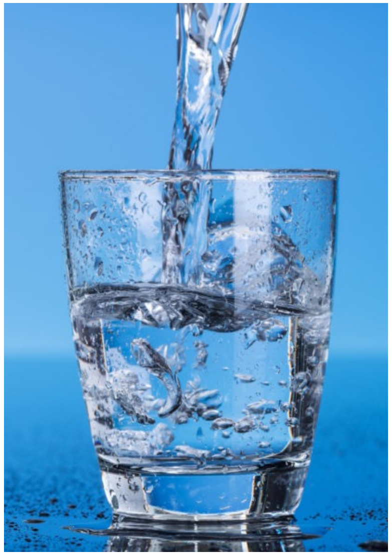

Separate salt and water, purify water

(2) Natural system

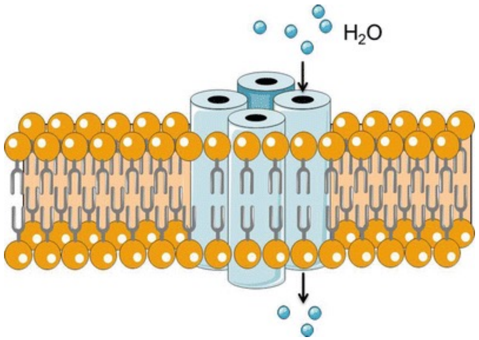

Aquaporin protein in all living cells

(3) Apply

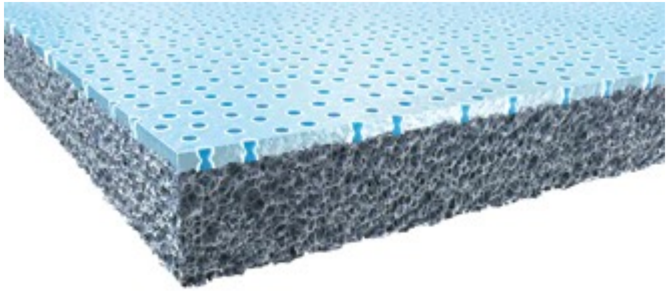

Membrane

AQUAPORIN

NANYANG TECHNOLOGICAL UNIVERSITY SINGAPORE

Benefits of bio-inspiration:

Realize functionalities that we cannot do traditionally, in much faster, efficient and sustainable manner.

We have seen the top-down process of applying bioinspiration:

Technical challenge → "Ask Nature"

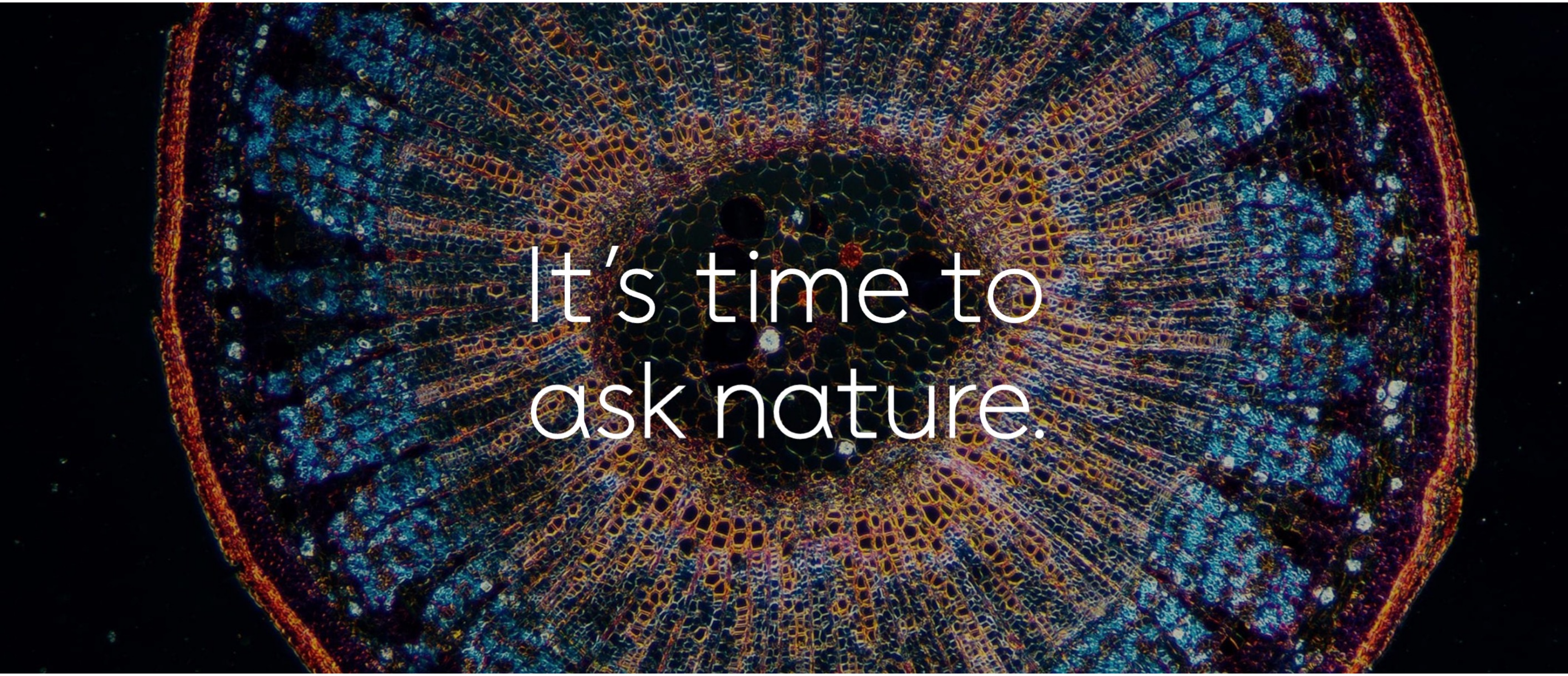

Asknature.org

We have seen the top-down process of applying bioinspiration:

Technical challenge → "Ask Nature"

There is also the bottom-up process :

Discovery in biology → Developing new technology

Applying the bioinspiration process – activity 3

Let’s look at some examples
from our research

Example of Venus inspired-inspired materials

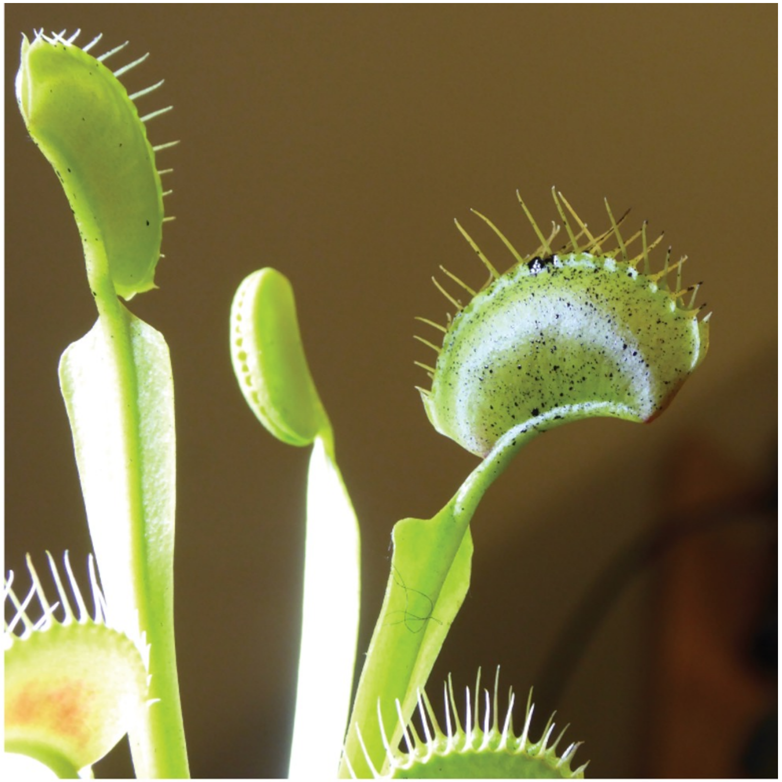

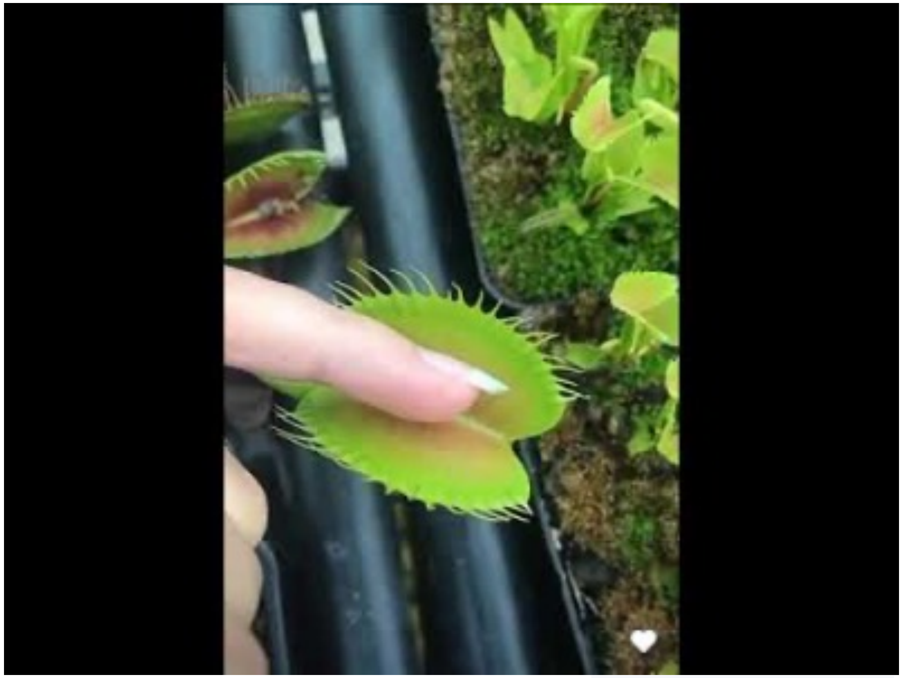

What mechanism or principle could we learn from the Venus Fly trap?

www.wooclap.com/SEENTU

NANYANG TECHNOLOGICAL UNIVERSITY SINGAPORE

Example of Venus inspired-inspired materials

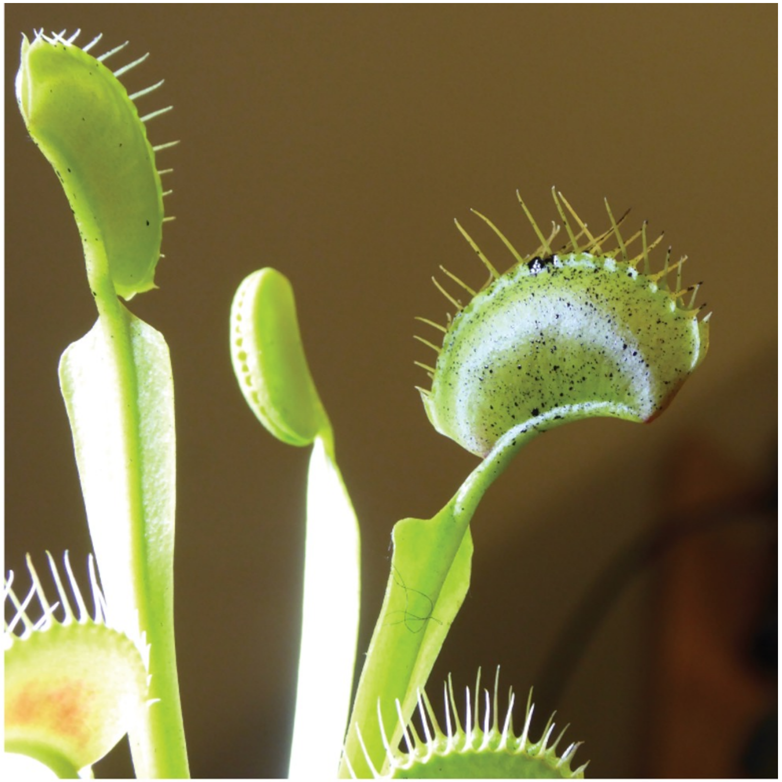

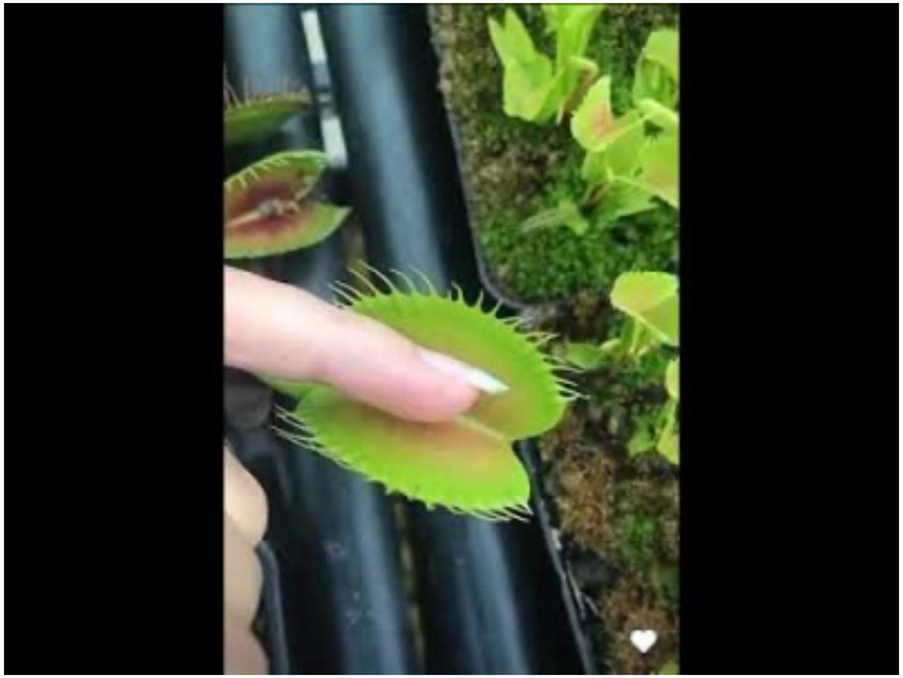

What mechanism or principle could we learn from the Venus Fly trap?

Fast morphing, complex shapes

Example of Venus inspired-inspired materials

Part of the fast closing of the Venus flytrap results from a mechanical instability which results in 2 positions: on and off.

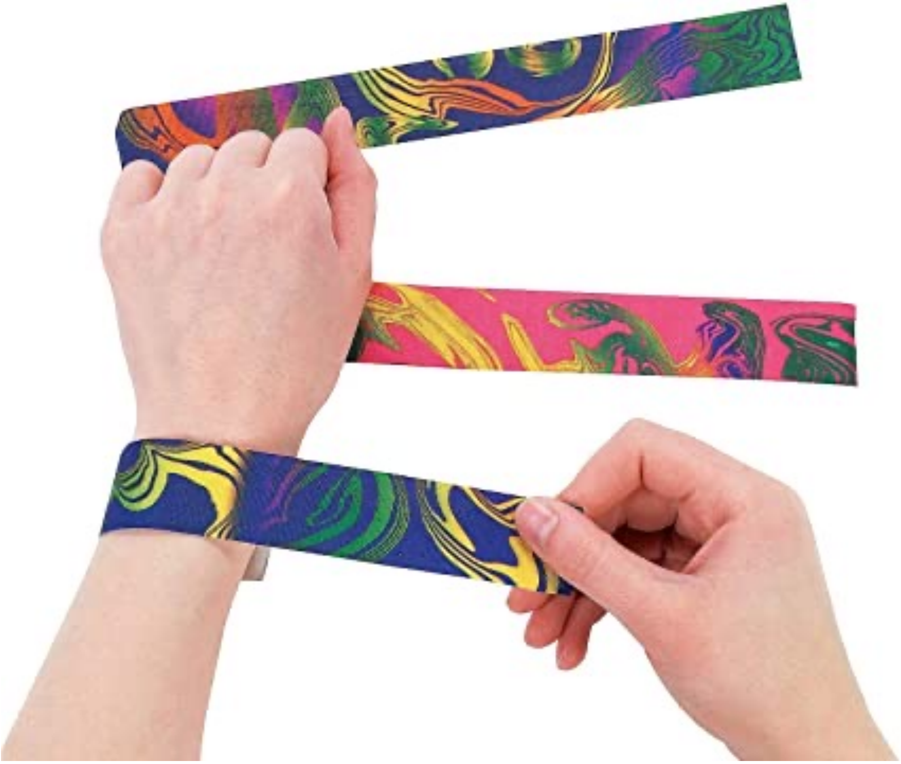

Example of Venus inspired-inspired materials

This is because there is energy stored in the material. This is called pre-stresses and they are due to the microstructure.

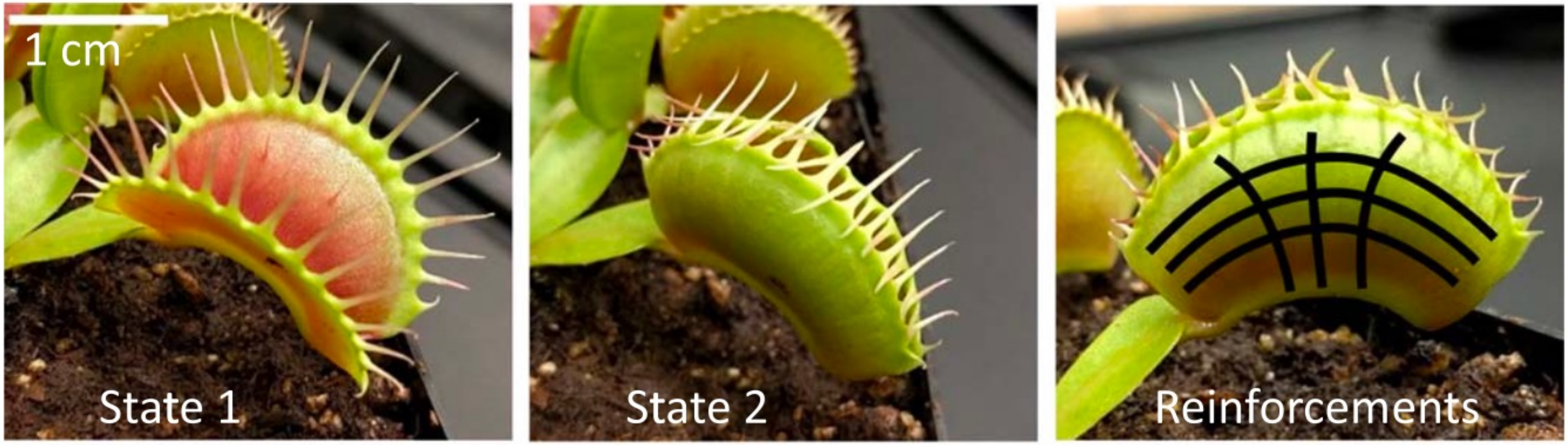

Example of Venus inspired-inspired materials

We are now applying the same principle in other materials systems.

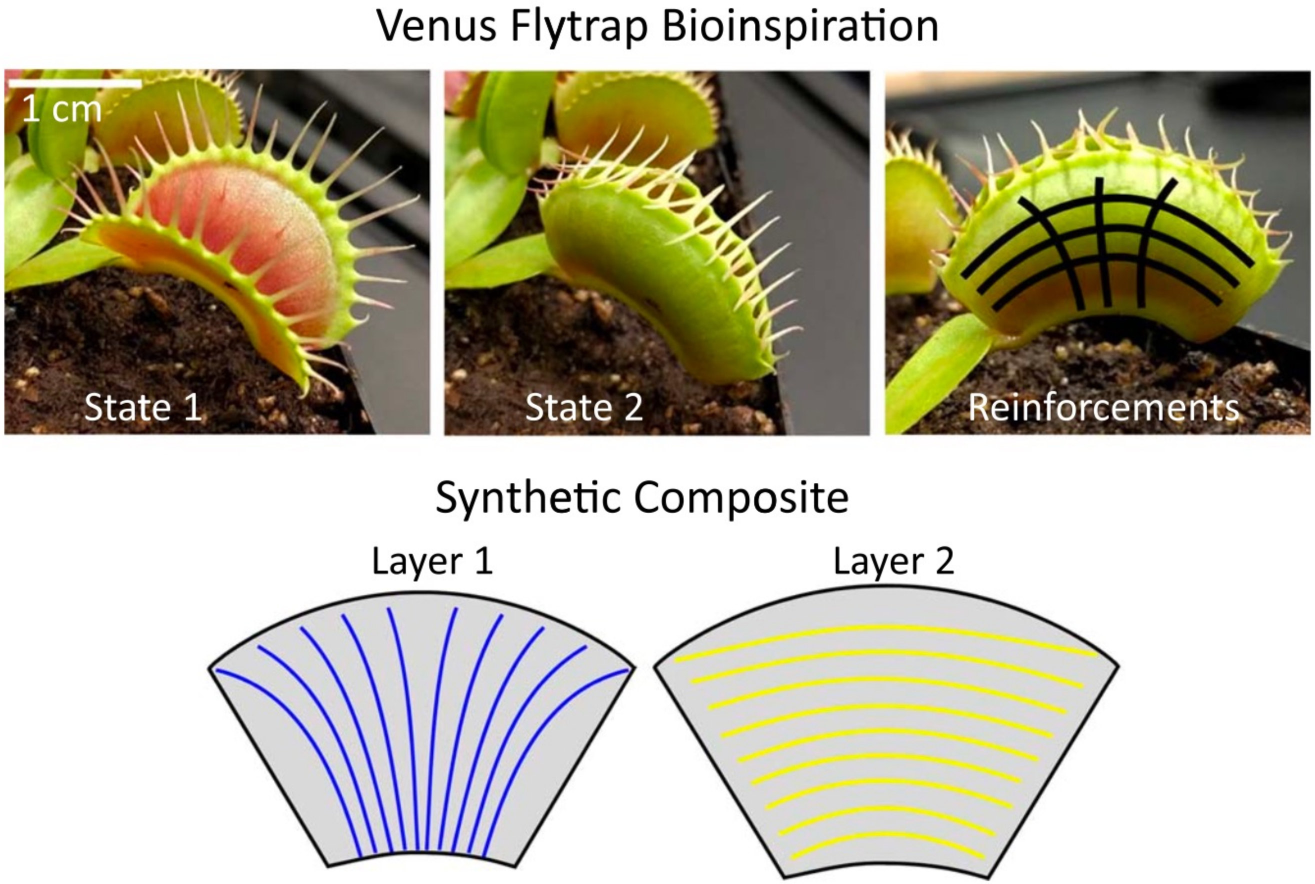

Example of Venus inspired-inspired materials

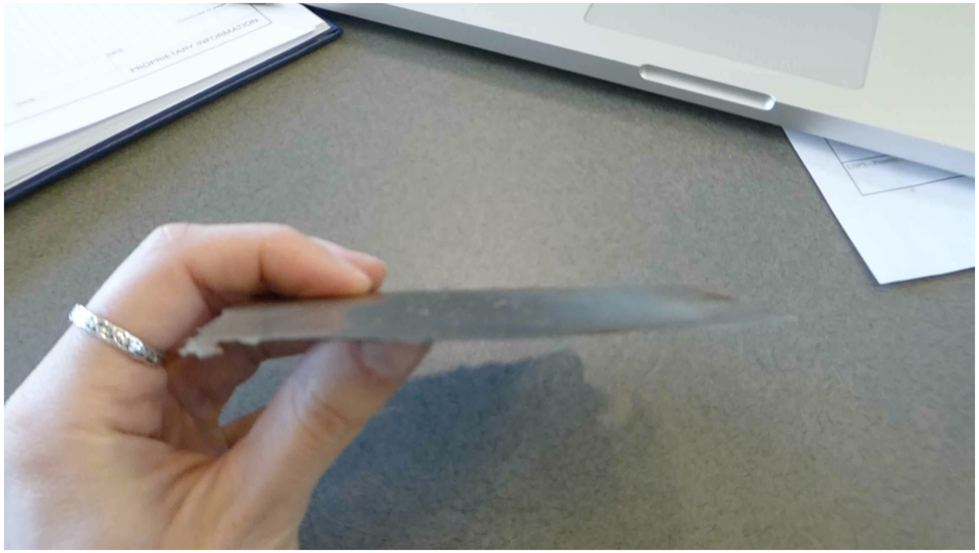

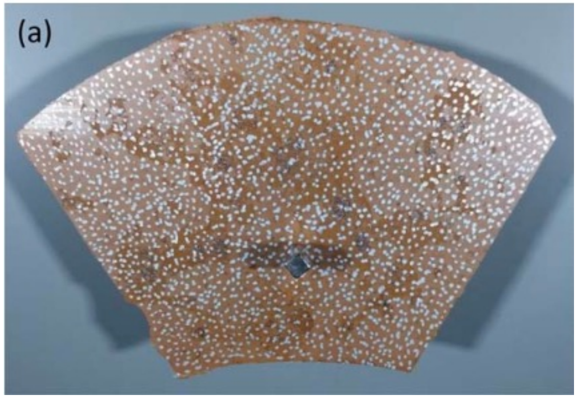

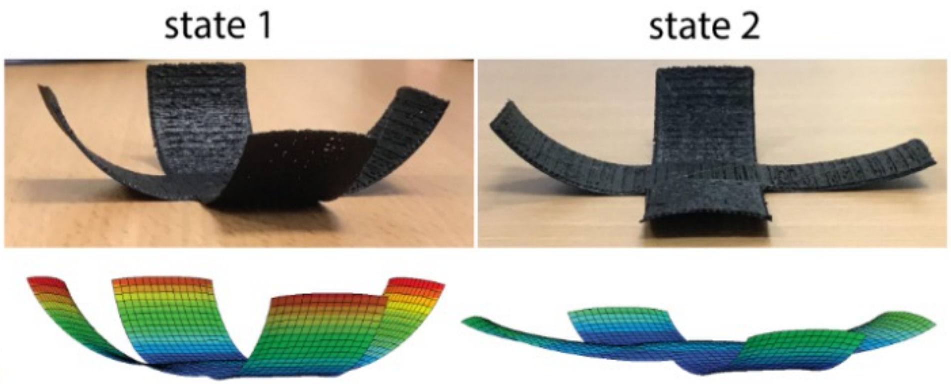

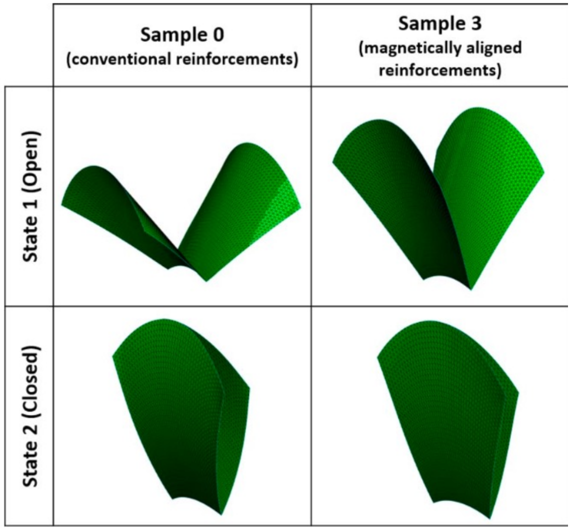

What could be an application for these kind of materials?

www.wooclap.com/BIOMIMICRY2

Example of Venus inspired-inspired materials

(1) Biological system

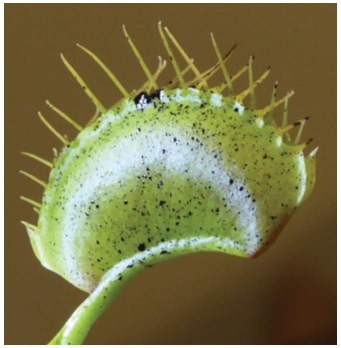

The Venus Flytrap that closes in less than 100 ms upon mechanical input.

(2) Understanding the principle behind

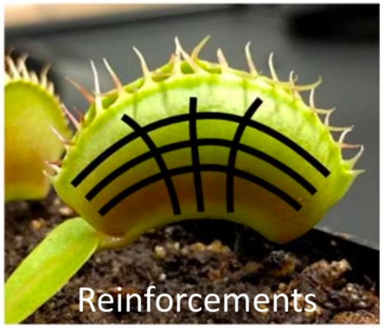

The leaves have perpendicular reinforcements that store energy and lead to bistability.

(3) Apply to innovate

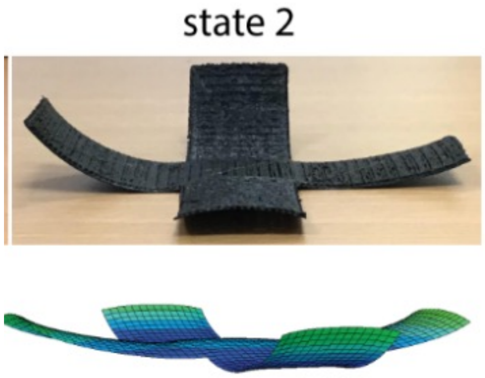

Create materials that are actuated in a very short time, repeatedly, in **<u>other compositions</u>**, shapes, for robotics, machines, etc.

Applying the bioinspiration process – activity 4

Each table has: (1) a biomaterial, (2) a testing procedure, (3) 2 envelopes

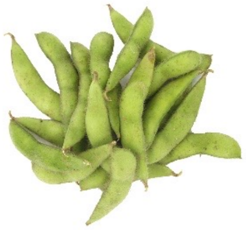

Edamame

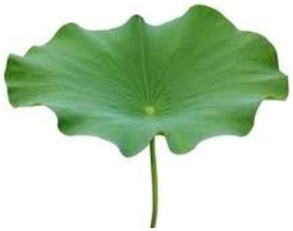

Lotus leaf

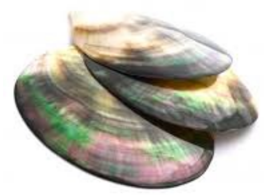

Seashell

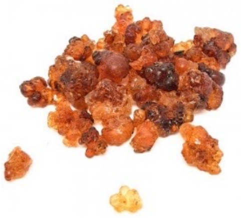

Peach gum

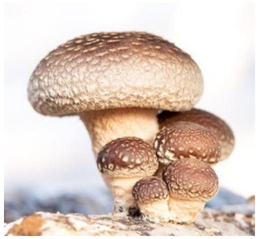

Mushroom

Download the PPT slides template. You will have to present the slides in 5-10 min at the end of the workshop

Group No.-

Name of the group members-

Preliminary thoughts

Insert Image of the object

Initial thought that came to your mind when you saw the object:

What you learned from the experiments

Insert Images/Videos

Brief description about what you did in the lab and what key concept you have learned.

Biomimicry/bioinspiration in application

For what application could the mechanism learned be used? Suggest a problem and how biomimicry from your object can be used to solve this problem?

- Problem/Aim:
- Natural System:
- Method of implementation:

Applying the bioinspiration process – activity 4

1. Discuss in groups about the object
2. Download the slides
3. Follow your facilitator to the lab. Take notes and pictures
4. Come back to the room to prepare the slides

It is …

Will have the presentation at 12…

Summary from our examples

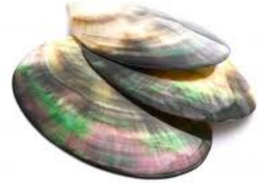

→ Aligned bricks → Strong & tough → Unbreakable ceramics

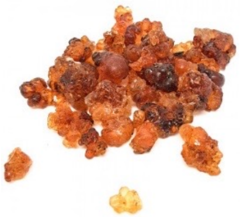

→ Chemical bounds → Sticky & self-healing → Adhesives

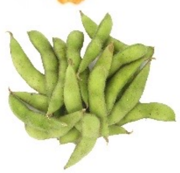

→ Bilayer → Morphing → Sensing

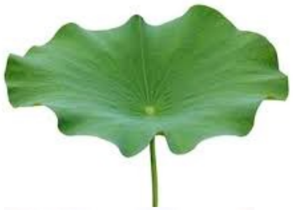

→ Nanopillars → Super-hydrophobic → Anti fouling

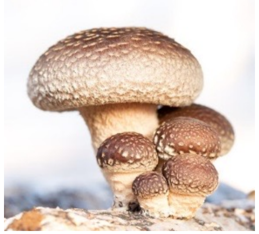

→ Gradient → No delamination → Orthopaedics

Strength and toughness challenge

Ashby plots

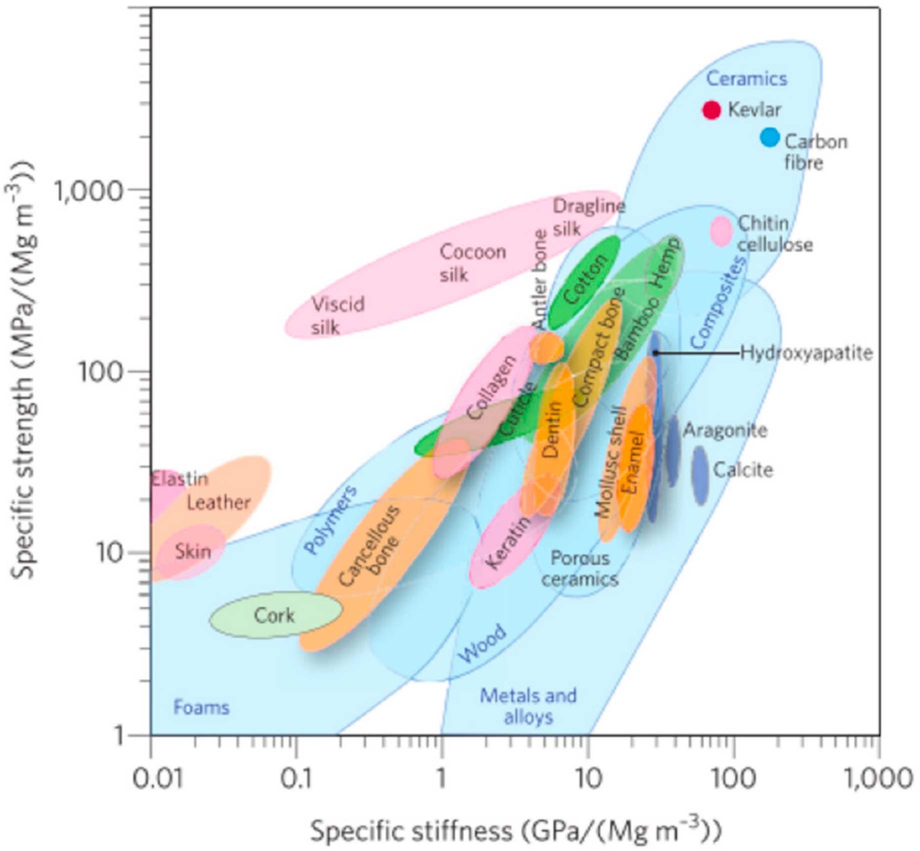

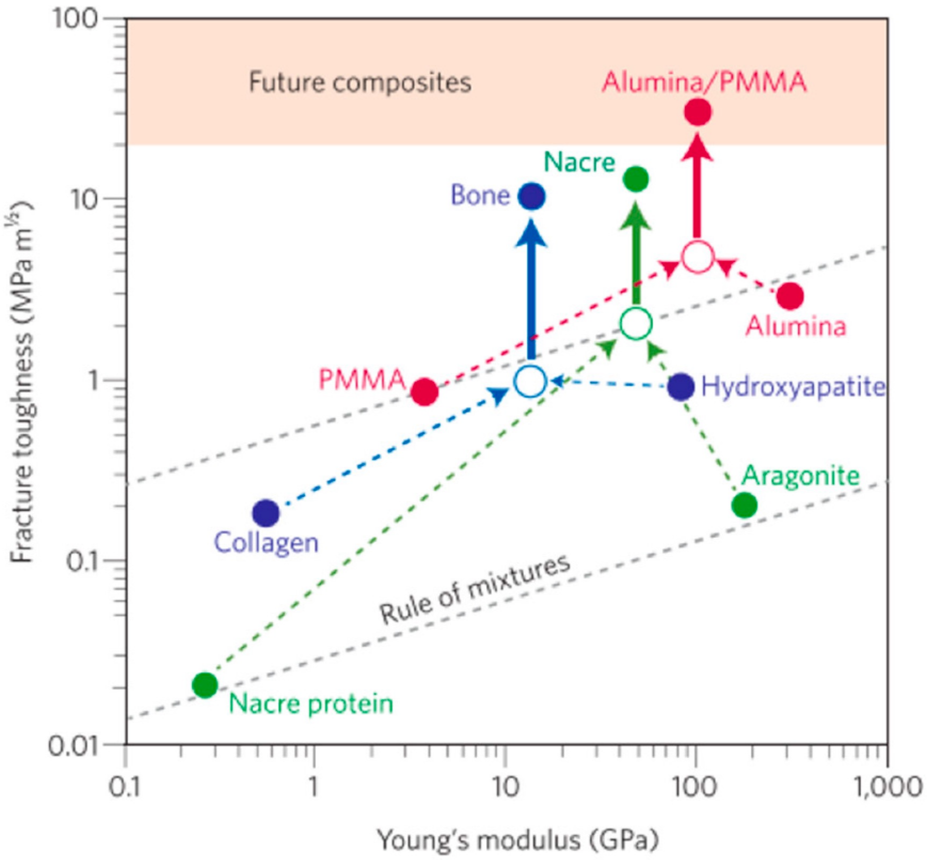

Morphing and manufacturing

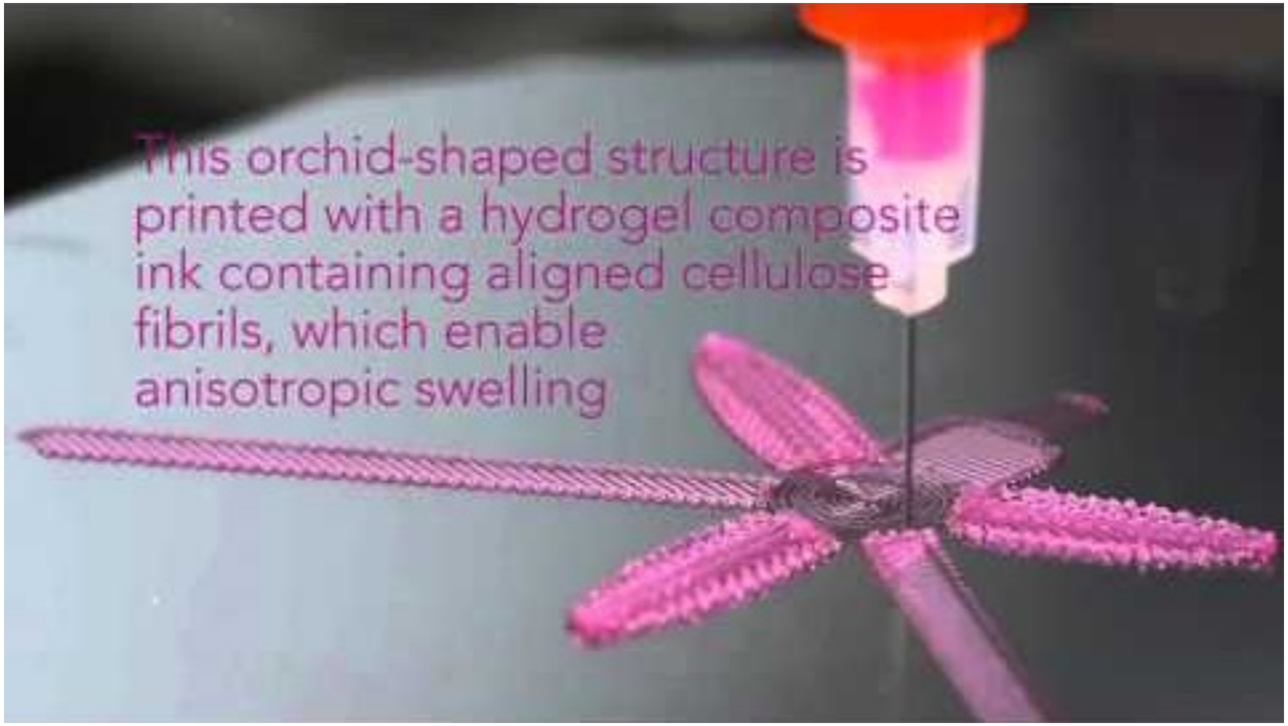

Problem of biofouling & self-cleaning

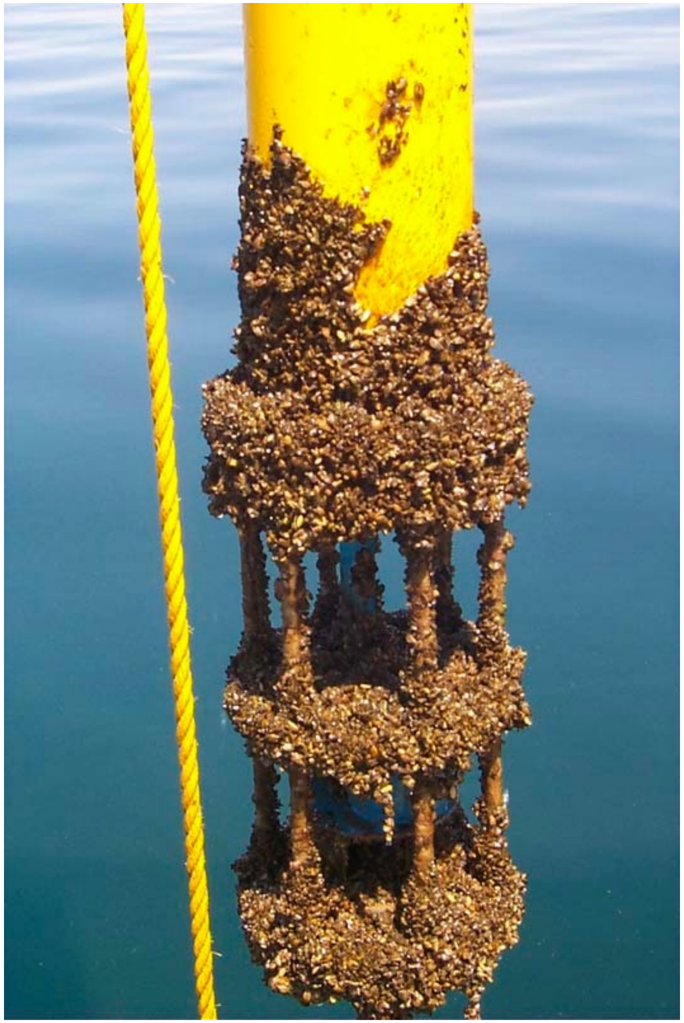

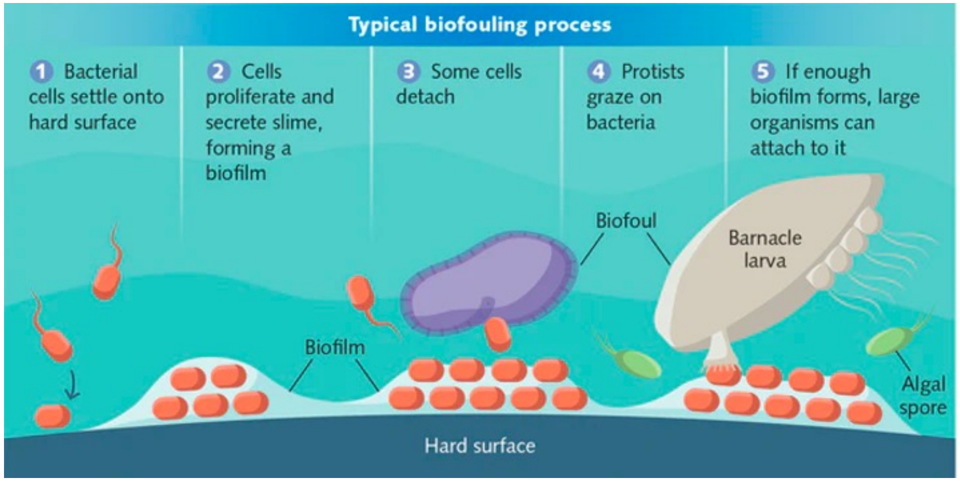

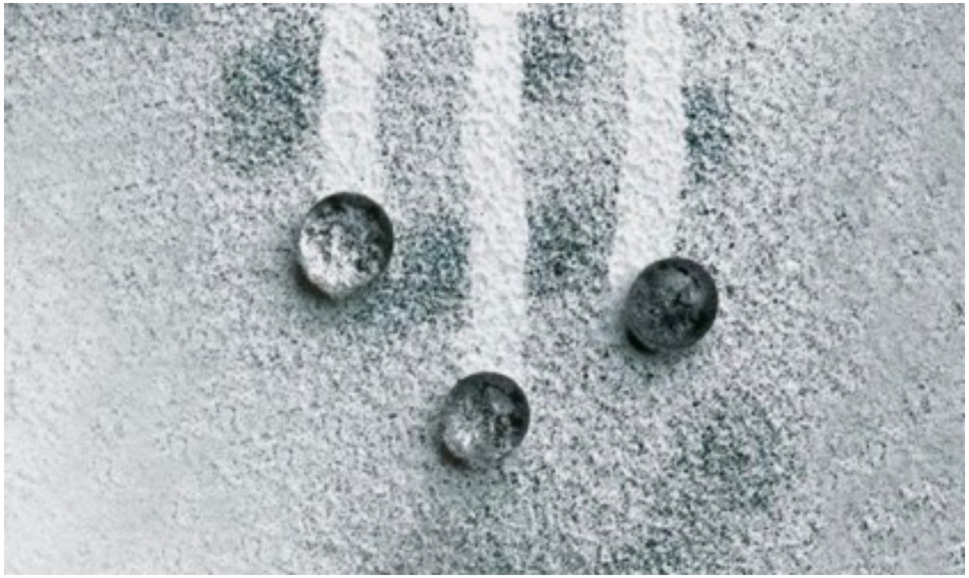

Problem of delamination

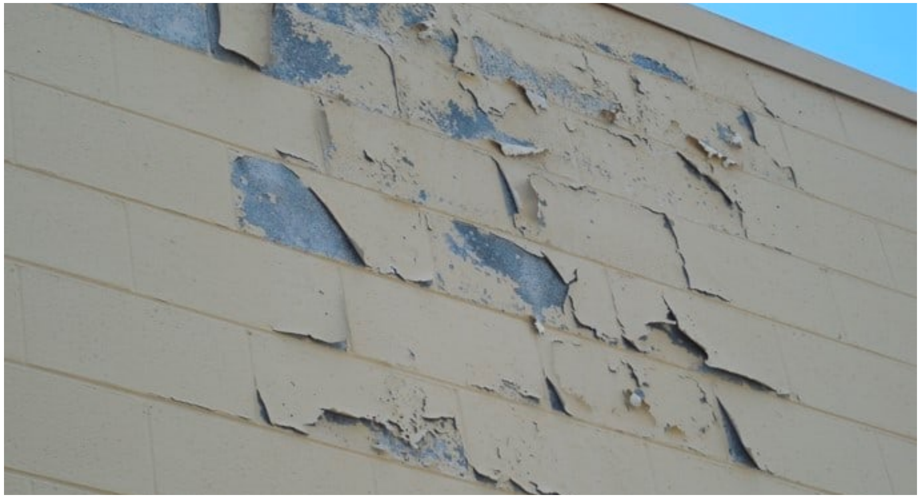

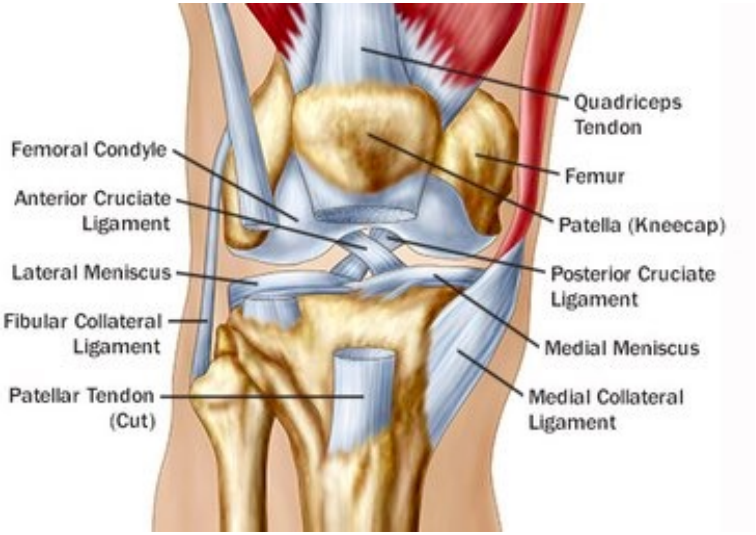

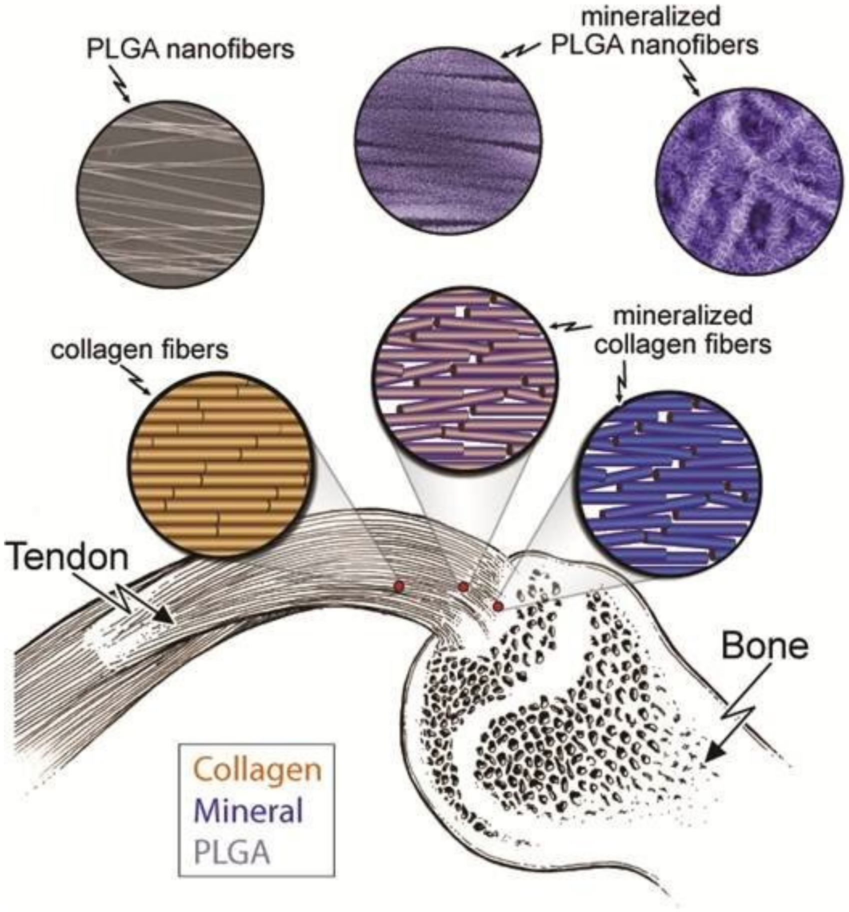

Biomimicry@Singapore

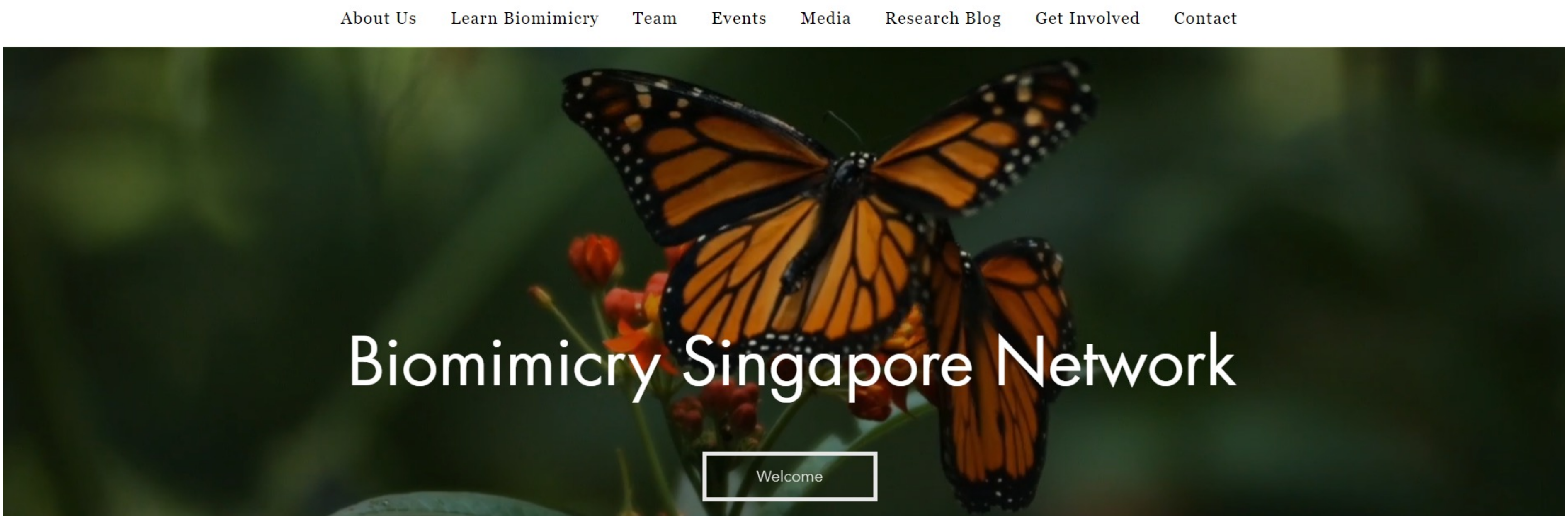

Bioinspiration@NTU

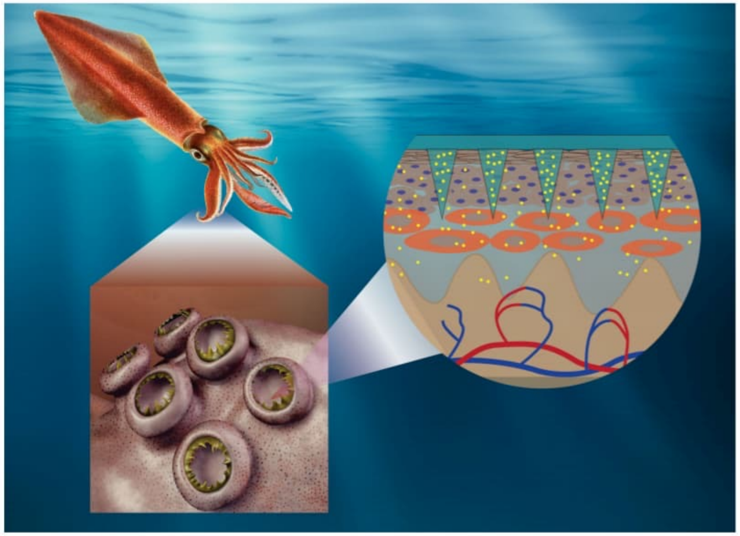

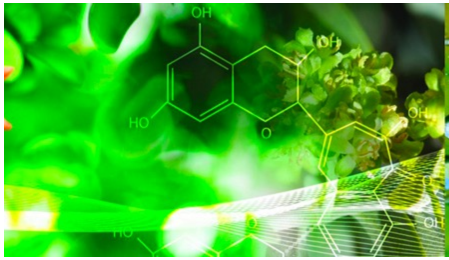

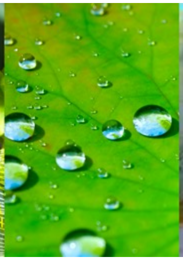

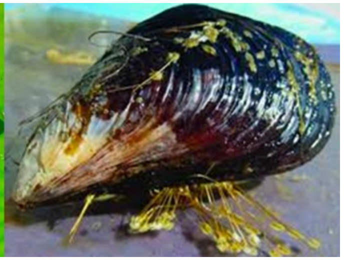

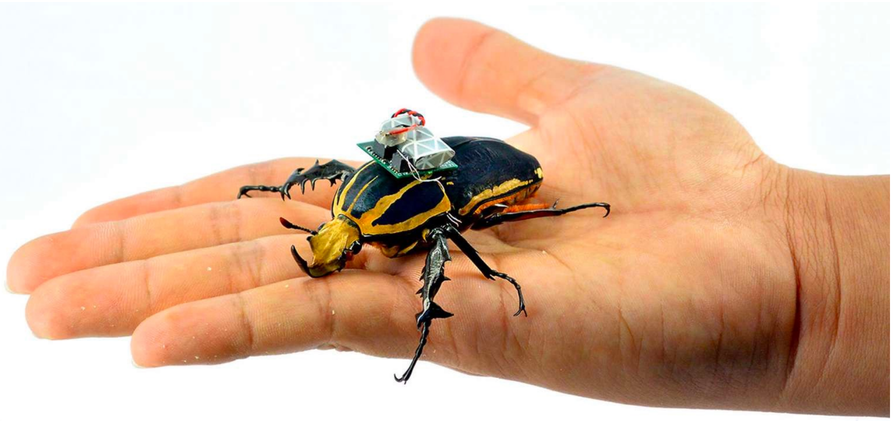

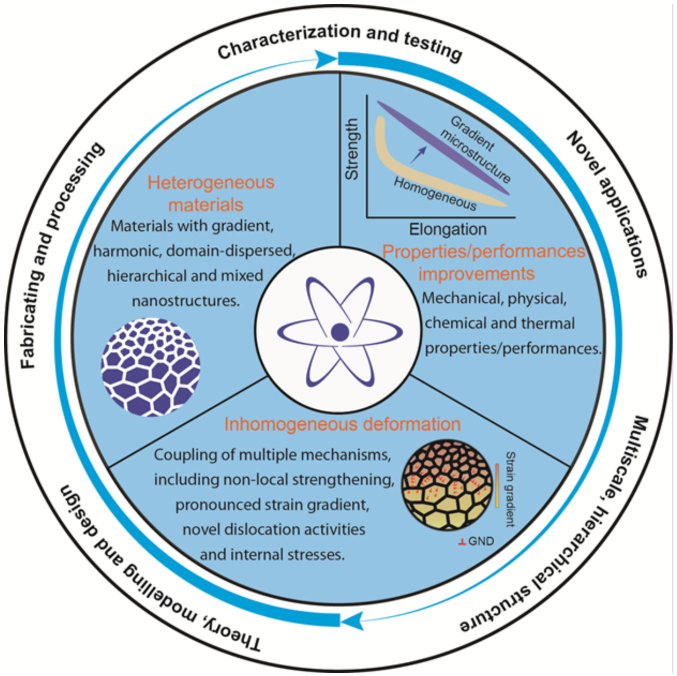

NANYANG TECHNOLOGICAL UNIVERSITY
SINGAPORE

Bioinspiration@Singapore

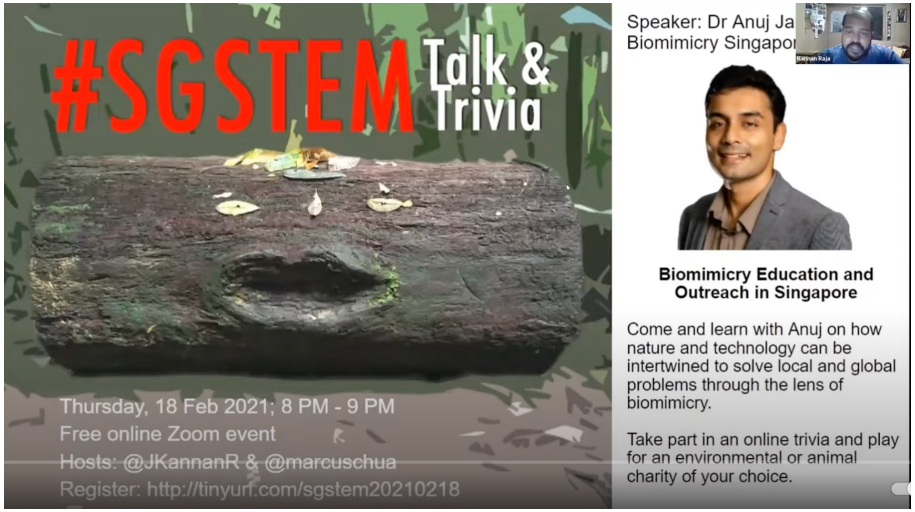

https://www.youtube.com/watch?v=9j6B8B6C8O0&ab_channel=SGSTEM

www.channelnewsasia.com › watch › building-nature-244...

Building With Nature - S1 - CNA

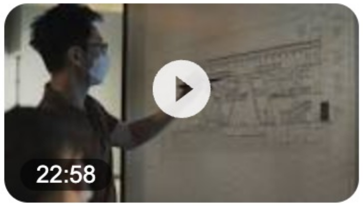

Drawing parallels between **Singapore's** greening journey and the futuristic design of the **Singapore** Pavilion ...

CNA · 1 month ago

NANYANG TECHNOLOGICAL UNIVERSITY SINGAPORE

Any Question?

Can you take some time to fill in the following?

Thank you!
